# TRESK is a key regulator of nocturnal suprachiasmatic nucleus dynamics and light adaptive responses

**DOI:** 10.1038/s41467-020-17978-9

**Published:** 2020-09-14

**Authors:** Tatjana Lalic, Aiste Steponenaite, Liting Wei, Sridhar R. Vasudevan, Alistair Mathie, Stuart N. Peirson, Gurprit S. Lall, M. Zameel Cader

**Affiliations:** 1 Translational Molecular Neuroscience Group, Weatherall Institute of Molecular Medicine, Nuffield Department of Clinical Neurosciences, University of Oxford, Oxford, UK; 2 Medway School of Pharmacy, University of Kent and University of Greenwich, Anson Building, Central Avenue, Chatham, Kent, ME4 4TB UK; 3 Department of Pharmacology, University of Oxford, Oxford, OX1 3QT UK; 4 Sleep and Circadian Neuroscience Institute (SCNi), Nuffield Department of Clinical Neurosciences, University of Oxford, South Parks Road, Oxford, OX1 3RE UK

**Keywords:** Cell biology, Neuroscience

## Abstract

The suprachiasmatic nucleus (SCN) is a complex structure dependent upon multiple mechanisms to ensure rhythmic electrical activity that varies between day and night, to determine circadian adaptation and behaviours. SCN neurons are exposed to glutamate from multiple sources including from the retino-hypothalamic tract and from astrocytes. However, the mechanism preventing inappropriate post-synaptic glutamatergic effects is unexplored and unknown. Unexpectedly we discovered that TRESK, a calcium regulated two-pore potassium channel, plays a crucial role in this system. We propose that glutamate activates TRESK through NMDA and AMPA mediated calcium influx and calcineurin activation to then oppose further membrane depolarisation and rising intracellular calcium. Hence, in the absence of TRESK, glutamatergic activity is unregulated leading to membrane depolarisation, increased nocturnal SCN firing, inverted basal calcium levels and impaired sensitivity in light induced phase delays. Our data reveals TRESK plays an essential part in SCN regulatory mechanisms and light induced adaptive behaviours.

## Abstract (web summary)

The suprachiasmatic nucleus (SCN) ensures rhythmic electrical activity that varies between day and night to determine circadian behaviours. The authors show that TRESK channels provide a feedback mechanism to maintain the SCN in the appropriate state for nocturnal light-induced behavioural changes.

## Introduction

The suprachiasmatic nucleus (SCN) is the primary circadian pacemaker synchronising internal circadian rhythms to the external day–night cycle. As well as intracellular clock gene rhythms, the SCN exhibits an endogenously generated electrical output, with higher spontaneous activity during the day and lower activity during the night^1^. It has been proposed that the nocturnal hyperpolarisation arises from changes in the functional expression of K^+^ channels^2^. By contrast, diurnal variation in a leak Na^+^ current encoded by the NALCN channel^3^ is considered to regulate daytime repetitive firing rates and resting membrane potential (RMP), along with input resistance. The antiphase cycling of Na^+^ and K^+^ conductances is an attractive model for driving daily oscillations of electrical activity in neurons, enabling clock neurons to convert their molecular time into circadian electrical output. Conversely, electrical firing feeds back to the molecular clock through calcium signalling, with the opening of voltage-gated calcium channels and internal calcium stores. Hence, the maintenance of appropriate intracellular calcium levels is key for proper SCN functioning.

The two-pore domain K^+^ channel (K2P) family have been traditionally considered as ‘leak’ background currents, which are the major contributors to RMPs. However, K2P channels can exhibit much more complex behaviour, with voltage-dependent activation^4^ to potentially regulate depolarisation and repolarisation kinetics and thereby neuronal firing frequencies. Furthermore, they are responsive to a range of signals including G protein subunits and uniquely, in the case of TRESK (TWIK-related spinal cord K^+^ channel), respond to calcium; therefore, they could be viewed as signal integrators at the membrane^5,6^.

We sought to further investigate the role of TRESK, encoded by the gene *kcnk18*, in SCN function using wild-type (WT) and knock-out mice, previously used to investigate pain biology^7^. We found TRESK K2P potassium channels to be enriched in the SCN compared to other brain regions. Therefore, we employed the TRESK knock-out (TRESK^−/−^) mouse model to assess alterations in SCN electrical properties, molecular changes and behavioural output. Furthermore, we demonstrate that K2Ps are critically important for the correct cellular state and responses of the SCN, which has implications for the growing list of disorders that involve K2Ps and for novel pharmacological interventions that may target these ion channels^8,9^.

## Results

### TRESK shows rhythmic expression and function in the SCN

**Fig. 1:**
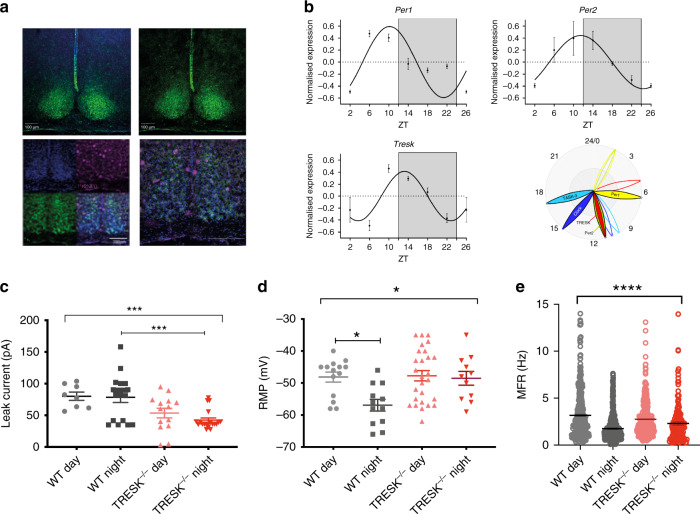
TRESK displays rhythmic expression and function in the SCN and is required for diurnal variation in resting membrane potential and spontaneous firing. **a** *Tresk* staining in the SCN using beta-galactosidase staining in TRESK^−/−^ mice (top panel) and *Tresk* mRNA in situ hybridisation (magenta colour) with co-immunohistochemistry with VIP (green) in the WT brain (bottom panel). Both staining methods confirmed abundant *Tresk* expression in the SCN. Scale bars for beta-galactosidase staining are 100 µm and for RNA scope images 200 µm. **b** RT-qPCR results of 24-h clock gene (*Per1* and *Per2*) as well as *Tresk* mRNA expression in WT mouse SCN, with *Tresk* and *Per2* mRNA levels peaking just before the night. Clock genes, *Tresk* and another K2P channel *Task-3* expression over 24 h with filled petals showing the peak and empty—nadir of measured expression. *n* = 3 SCNs for each time point. **c** In voltage clamp, SCN neurons were held at −60 mV before depolarisation to −25 mV and then subsequently ramped to −135 mV to isolate potassium leak current, in the presence of TTX. TRESK^−/−^ had reduced nocturnal potassium leak current compared to wild type (WT; day, *n* = 8; night, *n* = 18; TRESK^−/−^; day, *n* = 14; night, *n* = 20). Two-way ANOVA on log-transformed data; significant effect of genotype, *F*_(1, 55)_ = 13.08, *p* = 0.0006; interaction not significant. **d** Whole-cell, current clamp recordings of SCN neurons. RMP was depolarised by 9 mV during the subjective night in TRESK^−/−^ mice compared to WT mice. (WT; day, *n* = 14; night, *n* = 12; TRESK^−/−^; day, *n* = 27; night, *n* = 11). Two-way ANOVA, significant interaction between genotype and time, *F*_(1, 60)_ = 4.215, *p* = 0.044). **e** Spontaneous neuronal firing recorded using a 256-channel MEA. Diurnal variation of firing rate lost in the TRESK^−/−^ mice. Mean firing rate (MFR) calculated by pooling data from all SCN electrodes (WT; day, *n* = 7; night, *n* = 8 animals; TRESK^−/−^; day, *n* = 7; night *n* = 5 animals). Two-way ANOVA on log-transformed data; significant interaction between time and genotype, *F*_(1, 1320)_ = 6.418, *p* < 0.01). All grouped data are mean ± SEM. *p < 0.05, ***p < 0.001, ****p < 0.0001. Source data are provided as a Source Data file.

The K2Ps are important regulators of membrane potential and have been suggested to have an important part in the nocturnal hyperpolarisation of the SCN^10,11^, although the K2P primarily responsible has yet to be identified. A role for TRESK in the SCN has not previously been suggested but using the TRESK^−/−^ LacZ reporter mouse^7^ we performed beta-galactosidase staining to indirectly assess brain regions likely to express TRESK. Coronal brain slices showed abundant and selective expression in the majority of SCN neurons (Fig. 1a top). Given the lack of reliable and specific mouse TRESK antibodies, we further validated our findings by performing in situ hybridisation with co-immunohistochemistry of VIP staining (Fig. 1a bottom). We found *Tresk* mRNA homogenously expressed in the SCN including in VIP+ neurons. *Tresk* was expressed uniformly across the majority of SCN neurons (Supplementary Fig. 1a–c) with a similar number of *Tresk* mRNA puncta in the dorsal and ventral regions (Supplementary Fig. 1d; 6.5 ± 0.57 and 5.6 ± 0.5 mRNA puncta, respectively; mean ± SEM, *n* = 30 cells per region). This is, however, substantially lower than the expression observed in trigeminal ganglia, using the same approach (one-way analysis of variance (ANOVA), *p* < 0.0001, Supplementary Fig. 1d).

We next looked at the gene expression for *Tresk*, *Task-3* (another K2P), and core clock genes over 24 h. SCNs were harvested at 4-h intervals from WT mice entrained to a standard 12:12 light–dark (LD) cycle. Cosinor analysis indicated rhythmic gene expression for these genes (Supplementary Table 1). *Tresk* mRNA is clearly increased in the early night and continues to be upregulated through the night (Fig. 1b). *Tresk* and *Per2* show peak expression later than *Per1* but earlier than the peak of *Task-3*; with *Per1* and *Per2* expression patterns in line with the published work (Fig. 1b)^12^.

Given the diurnal *Tresk* expression, higher in the early subjective night, we investigated how this might affect the outward potassium leak currents in SCN neurons. We performed whole-cell patch-clamp recordings of SCN neurons from WT and TRESK^−/−^ mice using a ramp protocol to isolate the potassium leak current^7^. We found a significant reduction in leak current in the TRESK^−/−^ mice compared to WT (two-way ANOVA on log-transformed data; significant effect of genotype, *F*_(1, 55)_ = 13.08, *p* = 0.0006; interaction not significant, Fig. 1c and Supplementary Fig. 2). Interestingly, we did not observe any significant difference in potassium leak current in the WT mice between day and night, nor a significant difference between day and night for TRESK^−/−^ mice.

### TRESK is required for diurnal variation of SCN firing

We next examined the RMP in SCN neurons using patch-clamp recordings and observed the expected day to night regulation of RMP in WT animals, with the cells being more depolarised during the day (Fig. 1d). Strikingly in TRESK^−/−^ animals, there was a complete loss of diurnal regulation, with SCN neurons being constantly in a depolarised state (Fig. 1d, significant interaction between genotype and time, two-way ANOVA, *F*_(1, 60)_ = 4.215, *p* = 0.044).

Since there is no diurnal variation in overall leak current, then other mechanisms may principally determine the diurnal variation in RMP. To investigate whether the loss of TRESK might affect other leak currents, we measured gene expression of *Task-3* and *Nalcn* in the TRESK^−/−^ animals compared to WT. We found no significant changes, between genotypes, in the pattern of gene expression for *Task-3* but a substantive reduction in *Nalcn* at night (Fig. S3). Since NALCN produces an inward current with a depolarising effect, the significant reduction in the expression of this channel is likely a compensatory response to prevent any further depolarisation observed in TRESK^−/−^.

To assess how changes in leak current and RMP might affect the activity of SCN neurons, at the population network level, we used a 256-channel multielectrode array (MEA). Recordings were taken from acute slices containing SCN from the WT and TRESK^−/−^ mice during either the day (zeitgeber time 6 (ZT6)–10) or night (ZT12–18) (Fig. 1e). WT animals that had been maintained in 12:12 LD demonstrated the expected higher mean firing rate (MFR) during the day compared to the night (two-way ANOVA on log-transformed data; significant interaction between time and genotype, *F*_(1, 1320)_ = 6.418, *p* < 0.01). However, the diurnal pattern of firing was dampened in the TRESK^−/−^ mice, with a reduced difference between firing rate during the day compared to the night such that there was no significant difference. We note that the elevated daytime MFR in WT mice seems to be driven by a subset of electrodes, which is largely absent in nocturnal WT recordings. TRESK^−/−^ mice by contrast shows a wide variance in both the daytime and nocturnal recording, and at night, MFR is significantly higher in TRESK^−/−^ mice compared to WT mice. The MEA data parallel results from patch-clamp recordings of neurons in acute SCN slices, where the nocturnal spike rate is significantly higher in the TRESK^−/−^ mice compared to WT mice (two-way ANOVA, significant interaction between time and genotype, *F*_(1, 42)_ = 5.628, *p* < 0.05) and the former also have a higher proportion of spontaneously firing neurons, *χ*^2^_(1, 91)_ = 5.25, *p* value < 0.05) (Supplementary Fig. 4a, b).

### TRESK^−/−^ mice show no deficits in circadian entrainment

**Fig. 2:**
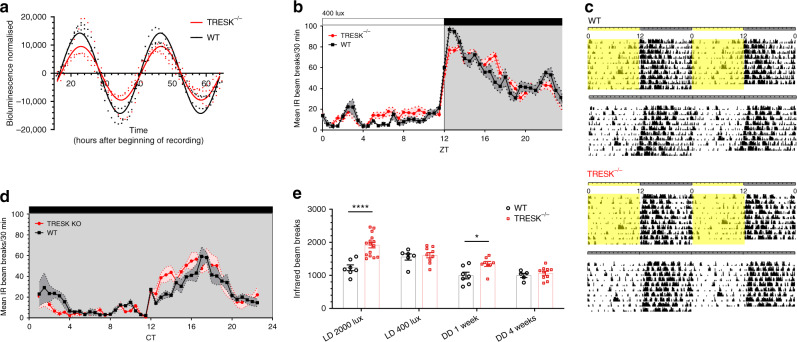
Loss of TRESK affects adaptive behavioural responses to light. **a** Bioluminescence recording of Per2::Luc WT and TRESK^−/−^ SCN slices shows maintained clock gene rhythmicity even in the absence of TRESK. Grouped and averaged over three animals for WT and three for TRESK^−/−^. **b** 24-H locomotor activity profile of animals housed under 400 lux illumination, resulting in similar locomotor activity pattern in both genotypes, although significantly increased activity during the day in TRESK^−/−^. **c** Representative actograms of WT and TRESK^−/−^ mice housed at 400 lux with 12:12 LD cycle and constant dark (DD). Light regime is indicated at the top of actograms, where yellow bars represent lights on and grey bars lights off. **d** 24-H free-running locomotor activity profile with TRESK^−/−^ mice displaying similar activity pattern as the WT. **e** 24-H infrared beam breaks during different illumination housing and free-running, showing light-dependant behaviour with greatest differences at 2000 lux. Housing in constant dark for 4 weeks eliminated light-induced behavioural differences. Two-way ANOVA for housing conditions (*F*_(3, 59)_ = 18.8, *p* < 0.0001) and genotype (*F*_(3, 59)_ = 20.2, *p* < 0.0001), Bonferroni’s multiple comparisons test for LD 2000 lux *p* < 0.0001, DD 1 week *p* = 0.036. All grouped data are mean ± SEM; shaded areas in **b** and **d** show SEM. *p < 0.05, ****p < 0.0001. Source data are provided as a Source Data file.

Next, we examined how changes in leak current, RMP and firing activity impact the molecular clock and behaviour. To measure the rhythmicity of the molecular clock in the absence of TRESK, TRESK^−/−^ mice were crossed with WT Per2::LUC mice resulting in TRESK^−/−^ Per2::LUC mice. Bioluminescence recordings of SCN organotypic cultures showed no differences in Per2 period indicating that the molecular clock is intact in the absence of TRESK (Fig. 2a). There was a 33.2% decrease in TRESK^−/−^ amplitude compared to WT controls (*p* = 0.0008, cosinor analysis, unpaired two-tailed *t* test to compare amplitudes).

Circadian behaviour was then assessed in WT and TRESK^−/−^ littermates by measuring locomotor activity with infrared (IR) sensors. TRESK^−/−^ mice showed normal entrainment to LD cycles (Fig. 2b) and showed clock-controlled endogenous locomotor activity in constant darkness (DD; Fig. 2c). Analyses of the circadian free-running period of mice in DD revealed no difference between WT and TRESK^−/−^ mice (circadian period, *τ* = 23.73 ± 0.03 and 23.87 ± 0.02, respectively (mean ± SEM); *p* > 0.05). Further detailed locomotor activity analysis shows no difference in the daily IR beam breaks but significantly increased daytime activity in TRESK ^−/−^ animals with delayed activity onset after the lights off (Supplementary Fig. 5a–f). Behavioural activity under DD did not show any differences in daily IR beam breaks; however, there was a significant increase in the amplitude of activity (Supplementary Fig. 5g–i). The essentially normal patterns of activity in 12:12 LD and DD conditions indicates that circadian entrainment is preserved despite the significant dampening in SCN diurnal variation of electrical activity in the TRESK^−/−^ mice.

### TRESK^−/−^ mice display dysregulated behaviour to light

**Fig. 3:**
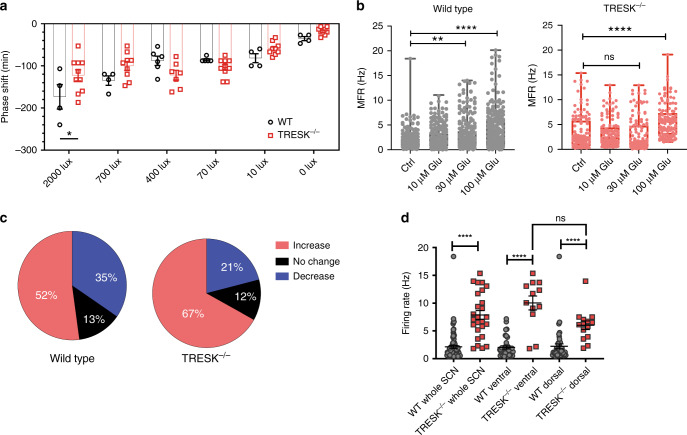
Loss of TRESK leads to altered glutamate responses of the SCN. **a** Sensitivity to free-running light exposure is diminished in TRESK^−/−^ mice. TRESK^−/−^ fail to show a light-driven dose–response relationship in circadian phase shifts, reaching saturation at 70 lux in contrast to wild-type littermates showing maximal responses at 2000 lux. 2000 lux light pulse *p* = 0.016 (Bonferroni’s multiple comparisons test). **b** Effect of glutamate on MFR of SCN neurons using a 256 MEA channel recording during ZT12-16 from WT or TRESK^−/−^ mice (WT; day, *n* = 7; night, *n* = 8 animals; TRESK^−/−^; day, *n* = 7; night *n* = 5 animals). Two-way ANOVA on log-transformed data; significant interaction between glutamate treatment and genotype, *F*_(3, 1077)_ = 15.15, *p* = 0.0001. **c** Activity of SCN neurons in response to glutamate, by separating the MEA electrodes to those that showed reduced firing (>10%), no change in activity (+/−10%) and electrodes showing increased firing (>10%), when comparing basal and 100 μM glutamate. TRESK^−/−^ compared to WT show a greater proportion of electrodes with increased activity after glutamate but a lower proportion with reduced activity. Chi-square test *χ*^2^ = (2,358), *p* = 0.0067. **d** Basal SCN firing rate in electrodes that subsequently reduced activity after 100 μM glutamate application in whole, ventral and dorsal SCN for WT and TRESK^−/−^. Significant differences found between wild-type and TRESK knockout animal of all three groups. Further significant difference found between the ventral and dorsal groups of TRESK^−/−^ mice. *p < 0.05, **p < 0.01, ****p < 0.0001. Comparison statistics: ANOVA one-way (whole SCN WT and TRESK^−/−^); ANOVA two-way log10(data) (ventral vs dorsal comparison). All grouped data are mean ± SEM. Source data are provided as a Source Data file.

We set out to establish the extent to which TRESK contributed to circadian photoentrainment. To this end, we measured phase shifts in free-running rhythm (activity in DD) following exposure to varying intensities of light. Light stimuli were administered 2 h following activity onset. This was extended to include a range of intensities allowing a dose–response relationship to be constructed for each genotype (Fig. 3a). Two-way ANOVA was significant for interaction and light intensity (*F*_(5,69)_ = 4.2, *p* = 0.002 and *F*_(5,69)_ = 25.7, *p* < 0.0001, respectively). WT mice exhibited a characteristic light intensity-dependent profile, with maximal phase shifts being observed at the highest light intensity (2000 lux). However, in TRESK^−/−^ mice behavioural shifts were saturated at 70 lux, with no significant increases observed through higher light intensities (Tukey’s multiple comparisons test). The most pronounced difference between genotypes was observed at 2000 lux, with WT mice having significantly greater responses relative to TRESK^−/−^ animals (*p* = 0.015, Bonferroni’s multiple comparisons test).

Furthermore, TRESK^−/−^ mice maintained under 12:12 LD but with 2000 lux daytime lighting rather than the usual 400 lux showed pronounced activity differences to WT mice. Under these conditions, WT mice showed significantly reduced locomotor activity during the subjective night, which the TRESK^−/−^ mice did not exhibit (Supplementary Fig. 6a). Activity in the later part of the subjective day was significantly increased under the 2000 lux LD cycle in TRESK^−/−^ mice compared to WT (two-way ANOVA, significant interaction between lighting conditions (*F*_(3,59)_ = 18.7, *p* < 0.0001) and genotype (*F*_(1,59)_ = 20, *p* < 0.0001), respectively). Increases in activity during the late night can also be seen from the actograms (Supplementary Fig. 6b). After 1 week in DD, TRESK^−/−^ mice maintained an increased activity profile, but after prolonged housing in DD (4 weeks), this locomotor activity matched that of WT littermates (Fig. 2d). This suggests a strongly light-dependent phenotype in TRESK^−/−^ mice.

Pupil measurements across different light intensities showed no abnormalities in TRESK^−/−^ mice, suggesting that all observed light effects are post retinal decoding and most likely at the level of the SCN (Supplementary Fig. 7). We also examined light-induced masking in the TRESK^−/−^ animals, first by placing TRESK^−/−^ and WT in the increasing photoperiod protocol, where the light period was increased by 2 h every 7 days (Supplementary Fig. 8a–d). TRESK^−/−^ mice were able to suppress their behaviour to the dark portion of the day with no differences to WT mice. The masking response was also evident when mice were given a 2-h light pulse between ZT14 and ZT16, and both TRESK^−/−^ and WT showed the ability to suppress their locomotor activity (Supplementary Fig. 8e). Hence, the pathways involved in masking appears intact in the TRESK^−/−^ animals.

### Lack of TRESK leads to altered SCN glutamate responses

Light information is relayed to the SCN, for entrainment and phase shifts, through the release of glutamate from terminals of the retinohypothalamic tract^13–16^. We investigated the effect of glutamate on acute MEA slices, with recordings during the night period, when light responses are expected to be maximal. During the lights-off period (ZT12–18), glutamate applied to acute slices in WT mice but not TRESK^−/−^ mice resulted in a graded dose-dependent increase in MFR (two-way ANOVA on log-transformed data, significant interaction between glutamate treatment and genotype *F*_(3, 1077)_ = 15.15, *p* = 0.0001, Fig. 3b). In TRESK^−/−^ mice, a response was only observed when the highest glutamate concentration was applied. The blunted dose–response curve to glutamate seems analogous to the blunted behavioural dose–response curve to light, except at the highest glutamate concentration.

After application of glutamate, we noted that, in some cases, neurons were showing increased activity and in other cases decreasing activity. To understand how loss of TRESK might affect these two main responses, we separated MEA electrodes by those which showed a ≥10% reduction of activity, electrodes with no change in activity (+/−10%) and electrodes showing ≥10% increase in activity. In WT animals, 52% of electrodes showed an increase in activity, 35% showed a decrease in activity and 13% showed no substantial change (Fig. 3c). In the TRESK^−/−^ animals, 67% of electrodes responded to glutamate by increasing activity, while only 21% responded by reduced activity. The differences between genotypes was highly significant, Chi-square test *χ*^2^_(2, 358_) = 10.0, *p* value = 0.0067. We also note that the population of electrodes responding by increased activity still show a relatively blunted glutamate dose-dependent increase in MFR in the TRESK^−/−^ compared to WT animals (Supplementary Fig. 9).

Interestingly, we found that the group responding by reduced activity in the TRESK^−/−^ had a significantly elevated basal MFR at 7.9 Hz compared to the corresponding group in WT mice, generating a basal MFR of 2.1 Hz (Fig. 3d). The basal firing rates of the remaining electrodes (those increasing activity or not changing activity after application of glutamate) was similar between WT and TRESK^−/−^ (Supplementary Figs. 9, 10). Hence the global increase in MFR at night in TRESK^−/−^ (Fig. 1e) may be driven, principally, by the subpopulation of neurons that are inhibited after application of glutamate. We next separated electrodes in the ventral retino-recipient region and the dorsal SCN. This revealed the electrodes with reduced activity after glutamate application and high basal MFR were largely being driven by those from the ventral region (two-way ANOVA, significant for interaction between genotype and SCN region, *F*_(1, 103)_ = 11.3, *p* < 0.001, Fig. 3d). Since the majority of SCN neurons in TRESK^−/−^ outside the retino-recipient region appear to have MFR with similar mean values to WT (although the differences between genotypes are statistically significant), including those of the dorsal SCN, this would likely explain the preserved diurnal behaviours in the knock-out mice, given the dorsal SCN is a key region in setting circadian output.

Overall this data reveals that loss of TRESK leads to fundamental alterations in the cellular responses of the SCN to glutamate, with more pronounced abnormalities in basal MFR in the ventral retino-recipient region. This may then be relevant to the dysregulated behavioural responses to light that we have observed.

### Loss of TRESK inverts SCN calcium dynamics

Glutamate released from RHT synapses leads to increased intracellular Ca^2+^, membrane depolarisation and increased in firing rate in the SCN neurons, through both activation of glutamate receptors (AMPA and/or *N*-methyl-d-aspartate (NMDA) receptors) and secondary activation of voltage-gated calcium channels^17–19^. Furthermore, the glutamate-induced Ca^2+^ influx underlies nocturnal light-evoked phase delays through downstream activation of transcriptional networks, including cyclic AMP-responsive element (CRE)-binding protein (CREB)^1^. Phosphorylated CREB is translocated into the nucleus where it can bind to CREs in the promoter regions of *c*-*Fos*, period 1 (*Per1*) and *Per2*, and drives transcription of these genes over a number of hours^20,21^.

**Fig. 4:**
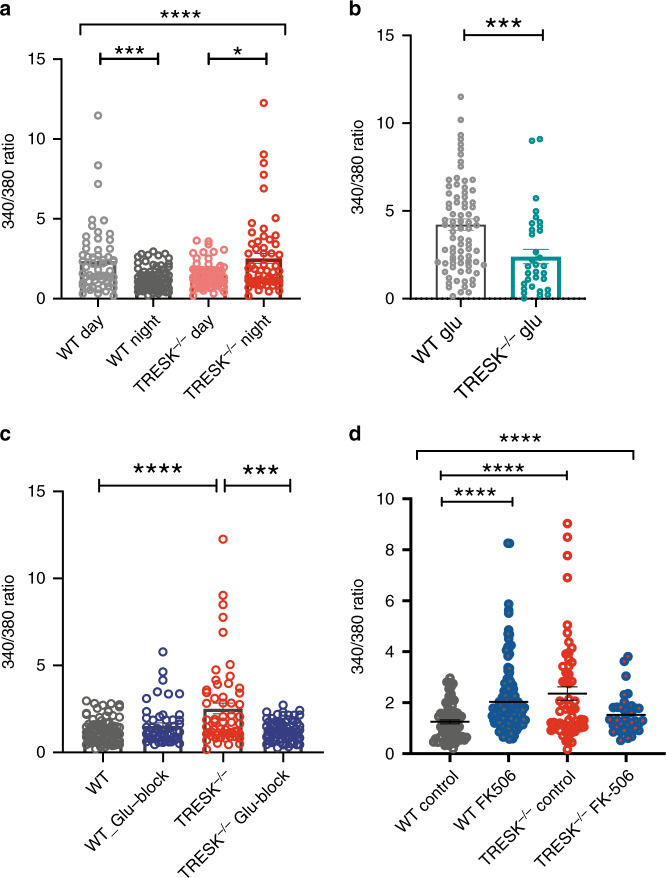
Loss of TRESK inverts SCN calcium dynamics, restored through blockade of glutamatergic activity. **a** Fura-2 calcium (Ca^2+^) imaging in the SCN neurons from WT and TRESK^−/−^ mice during the light and dark periods. Diurnal variation of basal Ca^2+^ relative ratios in the SCN neurons in the WT but this is inverted in the TRESK^−/−^. (WT; day, *n* = 94; night, *n* = 43; TRESK^−/−^; day, *n* = 35; night *n* = 26). Two-way ANOVA on log-transformed data; significant interaction between time and genotype (*F*_(1, 257)_ = 21.28, *p* = 0.0001). **b** Acute Ca^2+^ response of SCN neurons to 100 μM glutamate recorded during the subjective night from WT and TRESK^−/−^ mice. (WT; *n* = 83; TRESK^−/−^; *n* = 34). Welch’s *t* test, *p* = 0.0008. **c** Ca^2+^ ratio of SCN neurons, during the subjective night, after application of glutamate receptor (AMPA and NMDA) antagonists (DNXQ and D-AP5), results in nocturnal Ca^2+^ ratios restored back to wild-type levels (WT; *n* = 52; TRESK^−/−^; *n* = 71). Two-way ANOVA on log-transformed data; significant interaction between treatment and genotype, *F*_(1, 254)_ = 15.09, *p* = 0.0001. **d** Application of FK506 in WT mice during the subjective night increases the nocturnal Ca^2+^ ratio to a level, which is non-significantly different to TRESK^−/−^ Ca^2+^ levels during the night (WT; *n* = 146; TRESK^−/−^; *n* = 38). Two-way ANOVA on log-transformed data; significant interaction between treatment and genotype, *F*_(1, 309)_ = 24.17, *p* = 0.0001. All grouped data are mean ± SEM. *p < 0.05, ***p < 0.001; ****p < 0.0001.

We therefore explored Ca^2+^ dynamics in acute SCN slices using Fura-2 calcium imaging in the WT and TRESK^−/−^ during the light (ZT6–10) and dark periods (ZT12–18). Work by Colwell and others have reported an increase in intracellular Ca^2+^ during the day and a decrease during the night, matching the diurnal variation in MFR and RMP^1^. Ratiometric imaging of Fura-2 was performed to assess baseline calcium. We observed a higher basal calcium ratio during the day compared to night, as previously reported in WT mice (Fig. 4a). Surprisingly, in TRESK^−/−^ mice this is entirely reversed, and we see a significant elevated basal Ca^2+^ at night (two-way ANOVA on log-transformed data, significant interaction between time and genotype (*F*_(1, 257)_ = 21.28, *p* = 0.0001, Fig. 4a). Similar to the MEA data, we observed that a subset of cells were showing large variations in responses in WT mice during the day and this is largely absent at night. This pattern is inverted in TRESK^−/−^ mice. Low nocturnal basal calcium is considered to be important, as glutamate at night can cause phase-shifting responses through elevated intracellular calcium levels. When we applied glutamate in the dark period to the slices, we found that the acute Ca^2+^ response was significantly lower in TRESK^−/−^ compared to WT, due to the already higher basal Ca^2+^ levels (one-way ANOVA, (Welch’s *t* test) *p* = 0.0008; Fig. 4b). This likely explains the blunted phase-shifting responses we observed in TRESK^−/−^ animals, since light signals, through glutamate, are no longer able to efficiently induce the required calcium signal.

### TRESK prevents nocturnal glutamate-mediated calcium influx

A recent study has shown that astrocytes release glutamate at night as a key mechanism to suppress the SCN neuronal activity^22^. It was proposed that suppression of electrical activity was mediated through the effects of glutamate on presynaptic NMDA receptors of the GABAergic SCN neurons. We wondered, as an additional mechanism in post-synaptic neurons, whether TRESK may be a critical negative feedback mechanism that dampens glutamate receptor activity to then prevent an upward spiral in intracellular calcium levels. We reasoned that glutamate-induced Ca^2+^ entry and depolarisation should activate TRESK, since uniquely TRESK is the only K2P with a calcium-sensing domain.

We therefore applied glutamate receptor (NMDA and AMPA) antagonists (D(−)-2-amino-5-phosphonovaleric acid (D-AP5) [50 µM] and 6,7-dinitroquinoxaline-2,3-dione (DNQX) [12.5 µM]), respectively, to SCN slices from WT and TRESK^−/−^ mice and measured the chronic change in calcium levels (after 2–4 h). We found that the antagonists had minimal effects in WT, but in TRESK^−/−^ the nocturnal calcium levels were restored back to WT levels (two-way ANOVA on log-transformed data, significant interaction between treatment and genotype, *F*_(1, 254)_ = 15.09, *p* = 0.0001, Fig. 4c). We infer that, in TRESK^−/−^, the elevated intracellular calcium is principally arising from functional increased NMDA and AMPA glutamate receptor activity. We then examined further the involvement of TRESK in the dysregulated calcium dynamics by inhibiting calcineurin, the Ca^2+^-dependent activator of TRESK. Chronic application of FK506 (5 µM) in WT mice at night resulted in a significant increase in the intracellular calcium level nearly matching the intracellular calcium levels in TRESK^−/−^ mice at night (two-way ANOVA on log-transformed data, significant interaction between treatment and genotype, *F*_(1, 309)_ = 24.17, *p* = 0.0001, Fig. 4d). Importantly post hoc tests showed no significant differences between TRESK^−/−^ vs WT-FK506 treated and there was also no significant effect of FK506 on TRESK^−/−^. This therefore supports a key mechanism in the SCN, whereby the high level of nocturnal extracellular glutamate is prevented from upregulating SCN calcium levels and firing activity through a negative feedback loop involving calcium-dependent TRESK activation.

## Discussion

It is well established that the endogenous electrical excitation of the SCN is essential as an output. We found that the nadir in TRESK expression aligns with the daily increase in RMP and firing rate. Surprisingly, we did not observe diurnal variations in leak currents in SCN neurons, although outward leak current was overall reduced in TRESK^−/−^ mice compared to WT as expected. This suggest a more complex regulation of RMP than the expression of leak channels alone. Nevertheless, when TRESK is absent, there are dramatic changes in electrical characteristics. This includes the SCN being in a constantly depolarised state with elevated nocturnal firing rates in comparison to WT animals, suggesting an essential role for TRESK in SCN temporal outputs.

Despite such substantive electrophysiological changes, diurnal locomotor activities in TRESK^−/−^ mice are preserved, which is surprising since studies have shown clear correlation between diurnal SCN electrical activity and locomotor activity^23,24^. Other mechanisms may therefore operate to regulate diurnal locomotor activity, even when SCN diurnal firing patterns are dampened. Alternatively, or in addition, it is possible that a subset of cells within the SCN may yet maintain clear diurnal firing patterns despite the loss of TRESK. There is growing evidence that such ‘pacemaker’ cells may exist in the dorsal SCN to ensure initiation each day for synchronised circadian molecular oscillation over the whole SCN. Indeed, our data suggest that, in the absence of TRESK, the majority of SCN neurons do not in fact show elevated firing at night. Instead, only a minority subpopulation in the ventral SCN, which are characterised by reduced firing after application of glutamate, have a resting MFR of >10 Hz and largely drive up the global average at night. The firing rates in the dorsal SCN, which sets the SCN circadian output, by contrast appears to remain relatively intact. The importance of TRESK in SCN-dependent behaviours manifested when animals were required to adjust to both subtle and extreme changes to lighting conditions. The inability of TRESK^−/−^ mice to demonstrate an intensity-dependent shift in behavioural phase highlights the impact of this K2P channel in circadian adaptation to light. Above all the roles attributed to the SCN, the ability to adjust and keep time to LD cues is one of its most prominent features, and some may argue its crowning trait: the TRESK channel is a fundamental player in this process. Responding to light environments involves complex afferent pathways from cellular through to molecular events, centred on glutamatergic input from the retinohypothalamic tract. In the SCN of WT animals, there were different functional populations of neurons based on the response to glutamate, including a significant subgroup that respond with reduced activity. The loss of TRESK resulted in a significant shift, such that more neurons responded by increasing activity after addition of glutamate at the expense of neurons responding by reduced activity. Nevertheless, interestingly it was this type of neuron responding to glutamate with reduced activity, in the retino-recipient portion of the SCN, that showed very high basal MFR in the TRESK^−/−^. It will be interesting in future studies to investigate the functional importance of this subpopulation further.

**Fig. 5:**
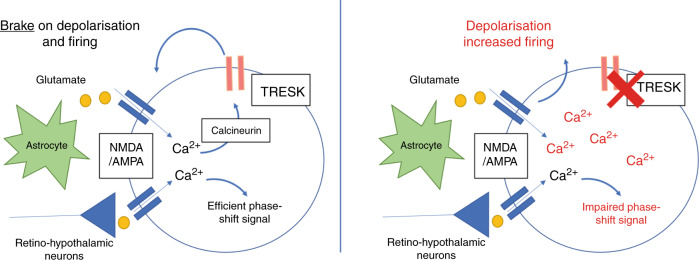
TRESK negatively regulates glutamatergic receptor signalling in the retino-recipient SCN. Under normal conditions (left panel), glutamatergic receptor activity induced by tonic activity from the retino-hypothalamic tract or nocturnal release from astrocytes results in membrane depolarisation and calcium influx. Activation of TRESK by calcium/calcineurin opposes the membrane depolarisation and ensures a hyper-polarised, low firing rate and low intracellular calcium state, which enables efficient light-induced phase-shifting signals. In the absence of TRESK (right panel), the negative feedback mechanism is absent leading to depolarised neurons, high basal firing rate and high intracellular calcium state.

Light in the early night results in a delayed phase shift through a glutamate-induced calcium influx. For an efficient signal, it is necessary that basal calcium levels in the SCN are lower at night than during the day. Hence, robust processes must exist that oppose any processes that might drive up nocturnal neuronal calcium. Recently, it has been shown that glutamate is released from astrocytes during the night, and our data suggest that TRESK is critical for preventing a wind-up of calcium levels that might occur in this context (Fig. 5). We find in the TRESK^−/−^ that diurnal calcium dynamics were completely inverted with significantly higher calcium at night than the day in the knock-out animals. The higher calcium is dependent on glutamate receptors since chronic application of NMDA and AMPA antagonists were able to normalise calcium levels, in comparison to vehicle controls. Since calcium-mediated calcineurin activation has previously been shown to activate TRESK^5^, we propose that TRESK is an essential component of the negative feedback regulation of glutamate receptor-associated calcium influx. In support, we show that the calcineurin inhibitor FK506 is able to elevate basal calcium levels in WT SCN to a similar level found in TRESK^−/−^ mice but has no effect when tested in TRESK^−/−^ mice. We cannot, however, exclude alternative or additional mechanisms leading to elevation of calcium after FK506 treatment, since calcineurin is known to have multiple effects in neurons. Nevertheless, the high basal calcium in TRESK^−/−^ reduces the acute glutamate calcium signal and explains the impaired phase shift observed in these animals. This data therefore reveals an important role for TRESK in providing a clear delineation of light responses between the day and night.

We have shown that TRESK is a key player in SCN function, regulating multiple aspects of SCN neurophysiology. Hence when TRESK is absent, increased nocturnal glutamate, from sources such as astrocytes, could activate glutamate receptors with no feedback mechanism to oppose membrane depolarisation and calcium influx. The resulting effect is that SCN neurons at night are relatively depolarised and hence have a higher MFR and higher basal calcium. These SCN neurons are then less able to respond appropriately to a pulse of glutamate that might occur with a light signal since basal calcium is already elevated. A predominant role of TRESK thus appears to be in maintaining the SCN in the appropriate state for nocturnal light-induced behavioural changes.

## Methods

### Animals

Young (P16–P23) WT C57BL/6 and TRESK^−/−^ mice (University of Oxford Breeding Unit) of both genders were used for in vitro electrophysiology, somal Ca^2+^ imaging and MEA recordings. Animals were group-housed in specific pathogen-free conditions on a 12:12 h LD cycle at 23 ± 1 °C with free access to food and water.

All behavioural testing was performed at Charles River Animal Facility using IR sensors (LuNAR™ PIR 360°, Risco Goup) and the data were collected using the Chronobiology Kit (Stanford Software Systems). Activity counts per minute were recorded and data were saved to the computer every hour. All mice were bred on a C57BL/6J background from heterozygous parents generating TRESK^−/−^ and WT animals. Male mice were housed individually in polypropylene cages with food and water available ad libitum.

All procedures complied with the UK Animals (Scientific Procedures) Act (1986) and were performed under a UK Home Office Project Licence in accordance with University of Oxford and University of Kent Policy on the Use of Animals in Scientific Research. The protocol was approved by the clinical medicine animal care and ethical review body, University of Oxford and University of Kent Policy on the Use of Animals in Scientific Research. This study conforms to the ARRIVE guidelines^25^.

### Electrophysiology

For whole-cell patch-clamp studies, 320-μm coronal slices containing SCN were prepared in ice-cold high-sucrose artificial cerebrospinal fluid (aCSF) containing 85 mM NaCl, 25 mM NaHCO_3_, 2.5 mM KCl, 1.25 mM NaH_2_PO_4_, 0.5 mM CaCl_2_, 7 mM MgCl_2_, 10 mM glucose and 75 mM sucrose after decapitation under isoflurane anaesthesia. Slices were then transferred to oxygenated aCSF (95% O_2_/5% CO_2_) containing 130 mM NaCl, 25 mM NaHCO_3_, 2.5 mM KCl, 1.25 mM NaH_2_PO_4_, 2 mM CaCl_2_, 1 mM MgCl_2_ and 10 mM glucose and then maintained at room temperature until recording.

During recordings, slices were superfused with aCSF saturated with 95% O_2_/5% CO_2_ at room temperature. Whole-cell patch-clamp electrodes (4–7 MΩ) were filled with an intracellular solution containing 120 mM K-gluconate, 10 mM KCl, 10 mM HEPES, 4 mM MgATP, 0.3 mM NaGTP, 10 mM Na-phosphocreatine and 0.5% biocytin. Slices were viewed with an upright microscope (Slicescope -Olympus BX51, Scientifica, UK), using an air objective (×2.5) to visualise the whole slice and locate SCN followed by water immersion lens (×40) and IR-DIC optics. Cells were typically visualised from 30 to 100um below the surface of the slice. The image was detected using a sensitive and fast camera (Photometrics Prime 95B sCMOS, Cairn, UK).

RMP and input resistance (*R*_input_) were measured immediately upon establishing whole-cell access. *R*_input_ was calculated by measuring the membrane potential change induced by hyperpolarising current injection from 20 to 100 pA from a holding potential of −60 mV. RMP and spontaneous firing was recorded in bridge mode while zero current was inputted (*I* = 0 mode). Intracellular recordings were obtained using a Multiclamp 700B amplifier and digitised at 10–20 kHz using Digidata 1440A acquisition board. While performing current-clamp recordings, a small amount of holding current (typically <−25 pA) was injected when necessary to keep the cell close to its initial RMP (−60 mV). To measure standing outward potassium leak currents, cells were held at −60 mV before depolarisation to −25 mV for 300 ms and then subsequently ramped to −135 mV over 550 ms. Outward currents were measured at the end of the 300-ms step. Baseline measurements were collected in the presence of 1 μM tetrodotoxin (TTX). Biocytin was included in the intracellular solution to allow post hoc visualisation and confirmation of cell identity and anatomical region. All data were analysed offline with Clampfit (pClamp 10), Neuromatic (http://neuromatic.thinkrandom.com) and custom-written software running within IgorPro environment.

### Ca^2+^ imaging

For ratiometric Ca^2+^ imaging, slices containing SCN were incubated in aCSF containing 5 µM Fura-2 AM and 80 µM pluronic acid (ThermoFisher Scientific) for 1 h at 37 °C, before being washed with aCSF (in mM 130 NaCl, 25 NaHCO_3_, 2.5 KCl, 1.25 NaH_2_PO_4_, 2 CaCl_2_, 1 MgCl_2_ and 10 glucose, pH 7.4, Osm 290–310). aCSF was perfused with the addition of drugs by a gravity-driven application system with software-controlled pinch valves. This allowed the drug application to be in a time-locked and hands-free manner. For somal imaging chronic drug application, drug or vehicle (aCSF) was added during the Fura-2 AM and pluronic acid incubation step. A ×40 objective, dichroic DC/T400lp and emitter DC/ET510/80m for infinity cube was used for calcium imaging. The fluorescence of fura2 was excited alternatively at wavelengths of 340 and 380 nm by means of a high-speed wavelength-switching device (Multistream Pro with NI USB-60001, Cairn, UK). Stabilised OptoLED power supply was used for controlled illumination and modulation of two-channel light-emitting diodes (LEDs).

Image analysis software (MetaFlour, Universal Imaging, PA, USA) allowed the selection of several ‘regions of interest’ within the field from which measurements are taken once every 1 s. Ratiometric 340/380 calculation was performed with a background subtraction. The 340/380 ratios were then analysed by measuring the average baseline in a user-defined time window using custom scripts in Matlab. The data were smoothed using robust local regression MATLAB function at 20%.

### Extracellular action potential recordings (MEA)

The SCN slices were placed on MEA array with 256 electrodes arranged in a 16 × 16 grid with inter-electrode distance of 100 µm. Signals from all electrodes on the 256 MEA array were collected simultaneously using USB-256MEA channel system (Multichannel Systems, Germany). Two-minute recordings were collected and repeated three times at 20 kHz. Channels within the SCN were identified visually, and spontaneous extracellular action potentials recordings from these SCN channels were discriminated offline using threshold-based event counting in custom-written scripts in IgorPro. Thresholds were typically set at 3.5× the baseline noise level, with typical signal amplitude between 20 and 75 µV. Single-unit discrimination was not routinely possible in these experiments. Custom-built MATLAB software was used to compare changes of activity at the highest dose of glutamate to baseline. If >10% increase was found, these neurons were grouped in the increased group. If >10% decrease was found, these were grouped in the decreased group. No change group contains neurons with 10% fluctuation above or below the baseline. Significance analysed with one-way ANOVA test.

### Statistical analysis

Data are represented as means ± SEM, and ‘*n*’ refers to the number of observations. The number of animals in each data set is ≥3. Comparisons for statistical significance were assessed by one- or two-way ANOVA and post hoc multiple comparisons *t* tests or unpaired *t* tests using GraphPad Prism. Data from MEA and calcium imaging had a significant positive skew and were not normally distributed. This data was therefore log normalised prior to ANOVA analysis.

### Drugs

TTX, D-AP5, DNQX, L-glutamic acid and FK506 (Tacrolimus) were purchased from Tocris Bioscience, Sigma-Aldrich or Stratech. Drugs were dissolved in distilled water or dimethyl sulfoxide to make stock aliquots at 1000–10,000× final concentrations and stored at −20 °C until required. Stock aliquots were diluted with oxygenated aCSF to final concentration immediately before use.

To assess the effect of glutamate on MEA activity of acute SCN slices, we bath applied different concentrations of glutamate (0, 10, 30 and 100 µM) with recording commencing 5 min after starting perfusion and continuing for 2 min. The same slice was used with successively increasing concentrations of glutamate.

### RNA scope staining with VIP

Brains from WT male mice were collected at ZT11.5, washed in phosphate-buffered saline (PBS) and fixed for 24 h in 4% formaldehyde solution at 4 °C. Brains were cryopreserved by dehydrating them in 15 mL falcon tubes containing 10%, 20% and 30% sucrose solutions (for about 24 h at each sucrose concentration, kept at 4 °C). The dehydrated brain was embedded in optimal cutting temperature compound (OCT) media and kept at −80 °C. Coronal 14 µm brain slices containing SCN were mounted on the SuperFrost Plus adhesion slides and stored at −20 °C. Slides containing SCN were selected for RNA scope staining. Slides were prepared for staining by washing them with OCT, baking at 60 °C for 45 min to prevent slices from detaching during the following steps and hydrogen peroxide incubation for 10 min was followed by 2× wash in the mili-q water. Slices were dried by washing them in 100% ethanol for 1 min and 60 °C 5 min baking. Immedge™ hydrophobic barrier pen was used to draw the barrier around slices, and after drying, slices were kept for 15 min in the hybridisation oven with the applied Protease Plus. Slices were washed two times in mili-q water. The rest of the steps were as indicated in the RNAscope® 2.5 HD Detection Reagent – RED User Manual, PART 2, Document Number 322360-USM with the exclusion of counterstaining the slides with haematoxylin. TRESK probe was used for *Tresk* mRNA staining, and positive as well as negative probes were used as controls.

After the signal detection step, slices were additionally stained with VIP and 4,6-diamidino-2-phenylindole (DAPI) (all of the steps were done in dim light, incubations were in the dark): (1) slices washed 2 × 5 min in 0.1% Triton X-100; (2) blocked for 1 h in 0.3% Triton X-100 containing 2% normal goat serum (NGS); (3) incubated for 48 h at 4 °C in 1:20 anti-VIP antibody (ab43841) diluted with 0.3% Triton X-100 containing 2% NGS; (4) washed 2 × 10 min, 1 × 30 min, 1 × 10 min in 0.1% Triton X-100; (5) 1:600 secondary antibody (goat anti-rabbit Alexa Fluor 488) incubation for 2 h in 0.3% Triton X-100; (6) washed 3 × 10 min in PBS; (7) 10 min DAPI incubation; (8) washed 2 × 10 min PBS, 1 × 10 min H_2_O; (9) slides were dried and mounted using ProLong™ Gold Antifade Mountant.

### Imaging

Stained slides were imaged using a Zeiss 780 inverted confocal microscope. Objective: LD LCI Plan-Apochromat ×25/0.8 Imm Korr DIC M27; Channels: 3, 12-bit; Image size: *x*: 603.64 µm, *y*: 603.64 µm; pinhole: Track 1 Ch1: 38 µm, Track 2 ChS1: 36 µm, Track3 ChS1: 38 µm. Filters: Track 1 Ch1: 410–488 (DAPI), Track 2 ChS1: 562–615 (RNAscope RED stain), Track3 ChS1: 490–544 (AVP). Lasers: Track 1 405 nm: 2.0 %, Track 2 561 nm: 2.0%, Track3 488 nm: 0.7000%, Rectangular grid tile scan with 15% overlap and 2× zoom. Images were processed using ImageJ.

### 24-H clock gene expression

Age-matched male mice were sacrificed by cervical dislocation at ZT2, 6, 10, 14, 18, 22 (3 mice per time point). During ZT2–10, the brains were removed under the room light, whereas during ZT14–22 the brains were removed under dim red light. To prevent SCN from signalling from the retina, eyes were immediately removed from animals sacrificed during ZT14–22. One-millimetre coronal brain slices were made using brain matrix, and SCN was dissected under the stereomicroscope. Dissected SCNs were immediately submerged in RNAlater (Sigma, UK), frozen on dry ice and then kept at −80 °C. RNA was isolated from tissue extracts using the RNeasy Mini Kit (QIAGEN, UK). The RNA concentration was measured using the Qubit RNA HS Assay Kit (ThermoFisher), and RNA was reverse transcribed by the Nanoscript Reverse Transcription Kit (Primerdesign, UK) with the final working cDNA concentration of 1 ng/μL. Twenty-microlitre reactions were prepared in triplicate in 96-well white plates (Alpha Laboratories, UK) consisting of 10 μL iTaq™ Universal SYBR® Green Supermix (BioRad), 1 μL 20× TaqMan gene expression assay, 6 μL RNase-free water and 3 μL cDNA (For TRESK expression 7 μL of cDNA were used). Reactions were run Applied Biosystems 7500 fast Real-Time PCR Systems.

20× TaqMan gene expression assays:

- Mm00501813_m1; *Per1*
- Mm00478099_m1; *Per2*
- Mm00455950_m1; *Clock*
- Mm01702237_m1; *Kcnk18*
- Mm02619580_g1; *Beta-actin*

Relative expression was calculated using 2^−∆Ct^, where ∆C_t_ is C_t_ (gene of interest) − C_t_ (reference gene—beta-actin)^26^. Linear detrending and normalisation to mean of the 2^−∆Ct^ values was done using BioDare2 online tool. Data were standardised using the equation:

1 $$x = \frac{{x - x_{{\rm{mean}}}}}{{x_{{\rm{max}}} - x_{{\rm{min}}}}}.$$

Graphs were plotted using GraphPad Prism 8, and standard sine wave was fitted to the data.

### Behavioural recording

Environmental lighting was administered using LED strips fitted above each row of cages providing equal irradiance to all animals. LD cycle was controlled by a programmed timer, and light intensity was adjusted using neutral density filters. Twenty-four-hour LD activity profiles were plotted by extracting data in 30-min bins over 9–10 days of activity recordings (excluding days on which cages were cleaned). Mean 24-h activity for each animal was calculated and plotted (mean + SEM). The phase angle of entrainment was calculated using the Actogram Phase Ruler available in the Chronobiology Kit Analysis tool by measuring the time difference (in min) between activity onset and the lights off. To get the individual IR beam breaks, data was extracted in 10-min bins and analysed in Excel. For each animal, the alpha period was determined based on the activity levels, even if the activity started/finished when the lights were on. Rho activity was the remaining activity during 24 h.

Free-running behavioural rhythms were measured from animals housed in 10 days of DD following stable entrainment to a LD cycle. ‘Eye-fit’ regression lines were fitted to activity onsets from actogram data by blinded experimenter. Data were extracted in 10-min bins for daily IR beam breaks or in 30-min bins for construing the 22-h behaviour profiles. Tau measurements and amplitude was calculated using Chi-Square Periodogram in the Chronobiology Kit Analysis tool.

To plot phase angle of entrainment, amplitude and tau, values for each day of measurement for each animal were used.

LD data: 400 lux TRESK^−/−^ (9), WT (6); 2000 lux TRESK^−/−^ (15), WT (7). DD data: TRESK^−/−^ (5), WT (5). Data were prepared using Microsoft Excel; statistical analysis and graphs were done using GraphPad Prism 7. All data are presented as means and standard errors.

### Circadian clock resetting with a light pulse

Animals were entrained to a 12:12 LD cycle before being placed into DD and allowed to free run for a minimum of 7 days. Previous LD cycles consisted of either 400 lux daylight or 2000 lux conditions. The time of activity onset was determined by analysing actograms the day before the light pulse was given, and the estimated time of activity onset was determined. Five-minute light pulses of varying light intensities were given 2 h following activity onset. Intensities consisted of 2000, 700, 400, 70 and 10 lux given using LED lights. Mice were kept in their home cages for the duration of the light pulse.

### Per2::Luc bioluminescence recordings

PER2 luciferase mice were crossed with TRESK^−/−^ mice and a new line was generated containing PER::Luc and with knocked down TRESK channel. For bioluminescence recordings, organotypic SCN cultures were used. Age-matched animals were sacrificed at the same time of the day (ZT4.5–6.5) over the 2 days. Data represent SCNs from three TRESK^−/−^ and three WT mice.

NMDG-HEPES recovery solution used for slicing was prepared on the day of experiment and used over 2 days. Prepared solution was filter sterilised in the hood and bubbled with carbogen gas mixture (95% oxygen: 5% carbon dioxide) before use. Mice were sacrificed by cervical dislocation, brain was sliced to 250-µm slices using compresotome and SCN from 2/3 slices per animal was dissected with the needle. Dissected SCNs were placed on the millipore membranes and then 24-well plates (1 SCN per millipore membrane) containing 500 µL of recovery media followed by 37 °C incubation in the oven for 1 h. Recovery media was prepared by adding 2.5 mM AP-V and 100 nM MK-801 to the recording media.

Recording media was prepared as a stock and kept in refrigerator for future use with B27, Luciferin and foetal bovine serum added just before use. For the bioluminescence recordings, 500 µL of recording media was added into white 24-well plate. Millipore membranes with SCN slices were transferred to the recording media, sealed with clear film and placed into 37 °C incubator for an hour, or the next day if more SCN slices had to be added. Prepared recording plate was then placed into TriStar^2^ S LB 942 Berthold microplate reader with 100 s of recording time every hour. MultiCycle software by ActiMetrics was used for data analysis and graphs were plotted using GraphPad Prism 8. To find the changes in amplitude, sine curve was fitted through the data and significance was checked using *t* test.

### Pupillometry

Five TRESK^−/−^ and 5 WT 12:12 LD entrained mice were dark adapted for 1 h before the pupillary measurements. Animals were hand restrained and the irradiant light provided by the xenon arc lamp (OptoSource High Intensity Arc, Cairn Research) was directed straight to the eye. Pupil recordings of the contralateral were done using charge-coupled device camera (Sony, Japan) capturing the image of the eye every 0.3 s.

All pupillary measurements were undertaken during the animals’ subjective day. Pupillary images were taken for 3 s followed by 60 s of image recording under a 480-nm filtered irradiant light. Light intensity was reduced by one log unit using neutral density (ND) filters 0ND–6ND (irradiance Log: 8.6–14.6 photons cm^−2^ s^−1^). Photon irradiance was calculated using equation:

2 $${\rm{Irradiance}} = \frac{P}{{h \,\times\, \frac{c}{\lambda }}} = \frac{{P \,\times\, \lambda }}{{h \,\times\, c}},$$

where

- *P*—light intensity measured in Watts,
- *h*—Planck’s constant (6.626 × 10^−34^ Ws^2^),
- *c*—speed of light (3 × 10^17^ nm s^−1^), and
- *λ*—light wavelength in nm.

The equation can be further simplified giving:

$${\rm{Irradiance}}\,({\mathrm{photons}}\,{\mathrm{cm}}^{-2}\,{\mathrm{s}}^{-1}) = P \times {\lambda} \times 5.03 \times 10^{15}.$$

Normalised pupil area was calculated by dividing fully constricted pupil areas (images captured after light exposure) by fully dilated pupil areas (images captured before light exposure) for each animal during all irradiances by using the ImageJ software (NIH, USA). Data were plotted using GraphPad Prism 7 with error bars representing SEM.

### Increasing photoperiod

Eight TRESK^−/−^ and 6 WT littermates were housed under IR sensors with 400 lux illumination during the light phase provided by LED strips fitted above the cages. Animals were entrained under 12:12 LD cycle with lights on 6:00–18:00 for 14 days. On day 15, the LD cycle was changed to 14:10 LD with lights on 5:00–19:00 (photoperiod was increased by adding 1 h to the dawn and 1 h to the dusk) and it was maintained for 7 days. Two-hour increase in photoperiod was performed weekly resulting in 7-day photoperiods of 12:12, 14:10, 16:8, 18:6, 20:4 and 22:2 LD with the last photoperiod being in constant light. Days 3–5 after the change in photoperiod were used for the analysis of phase angle of entrainment, alpha period measurements and activity level during the dark phase of the day.

### Masking

Seven TRESK^−/−^ and 7 WT mice were housed under 12:12 LD cycle with 400 lux illumination during the light phase provided by LED strips fitted above the cages. Locomotor activity was recorded using IR sensors. Once a week, mice received an extra 2-h light pulse at ZT14–16. Baseline activity was measured from 2 days before the light pulse. Presented data are the average of 3 days with IR beam breaks data being extracted in 1-min bins.

### *Tresk* mRNA puncta assessment

RNA scope images were digitally zoomed to better visualise pink *Tresk* mRNA puncta. SCN regions were identified from VIP and DAPI staining helping to identify the outline of the SCN. The lowest part of the SCN was considered ventral and the uppermost part dorsal. None of the neurons from the middle of the SCN were used for analysis. Puncta were manually counted of 30 dorsal and 30 ventral randomly selected neurons on both sides of the SCN nuclei. Statistical analysis was done using unpaired *t* test and graph was plotted using GraphPad Prism 8.3.

### TASK-3 expression in the SCN

Mice were housed under the 12:12 LD cycle with 400 lux light intensity during the light period of the day. Four TRESK ^−/−^ and 4 C57BL/6J mice were sacrificed at ZT6 and ZT14 by cervical dislocation. Brains were chilled for 1 min in the ice-cold PBS, sliced into 1 mm sections using Coronal Brain Matrix and SCN was micro-dissected using a needle. Dissected SCNs were snap frozen on dry ice and then kept at −80 °C until processing.

RNA extraction and reverse transcription was performed as already described previously (24-H clock gene expression section). Twenty-μL reactions were prepared in duplicate in 96-well white plates consisting of 10 μL SYBR™ Green PCR Master Mix (Applied Biosystems, 4309155), 2 μL nuclease-free water, 0.9 μL 10 μM of each forward and reverse primers and 3 μL cDNA. PCR was performed using ROCHE LightCycler 480 instrument using the following protocol: pre-incubation (95 °C for 2 min); Amplification (50 cycles × 95 °C for 15 s, 60 °C for 60 s); melting curve (continuous: 95 °C for 15 s, 60 °C for 60 s, 95 °C for 30 s, 60 °C for 15 s).

Relative expression was calculated using 2^−∆Ct^, where ∆Ct is Ct (gene of interest) − Ct (reference gene—beta-actin)^26^. Data were rescaled to have values between 0 and 1 by using the formula:

3 $$x_{{\rm{new}}} = \frac{{x - x_{{\rm{min}}}}}{{x_{{\rm{max}}} - x_{{\rm{min}}}}}.$$

Graphs were plotted using GraphPad Prism 8.3. Data were analysed using two-way ANOVA with Tukey’s multiple comparisons test. All values are plotted as mean ± SEM.

Primers:

- *Task3*: Forward Primer 5′–3′ – AGCGGCAGAACGTGCGTACC; Reverse Primer 3′–5′ – AGGTGTTCATGCGCTCGCCC
- *Beta actin*: Forward Primer 5′–3′ – ACCAACTGGGACGATATGGAGAAGA; Reverse Primer 3′–5′ – CGCACGATTTCCCTCTCAGC

### Reporting summary

Further information on research design is available in the Nature Research Life Sciences Reporting Summary linked to this article.

## Supplementary information

Supplementary Information

Reporting Summary

## Data Availability

Source data are provided as a Source Data file. Data are available upon request.

## Source data

Source Data

## Code availability

Code is available upon request.
