# Supplementary material for: TRESK is a key regulator of nocturnal suprachiasmatic nucleus dynamics and light adaptive responses
Source: Nat Commun. 2020 Sep 14;11:4614. doi: 10.1038/s41467-020-17978-9 (PMC7490422; doi:10.1038/s41467-020-17978-9)
Supplement: Supplementary file 1 — Supplementary Information [file 41467_2020_17978_MOESM1_ESM.pdf]

Supplementary Information

TRESK is a key regulator of nocturnal suprachiasmatic nucleus dynamics and light adaptive responses

Lalic et al.

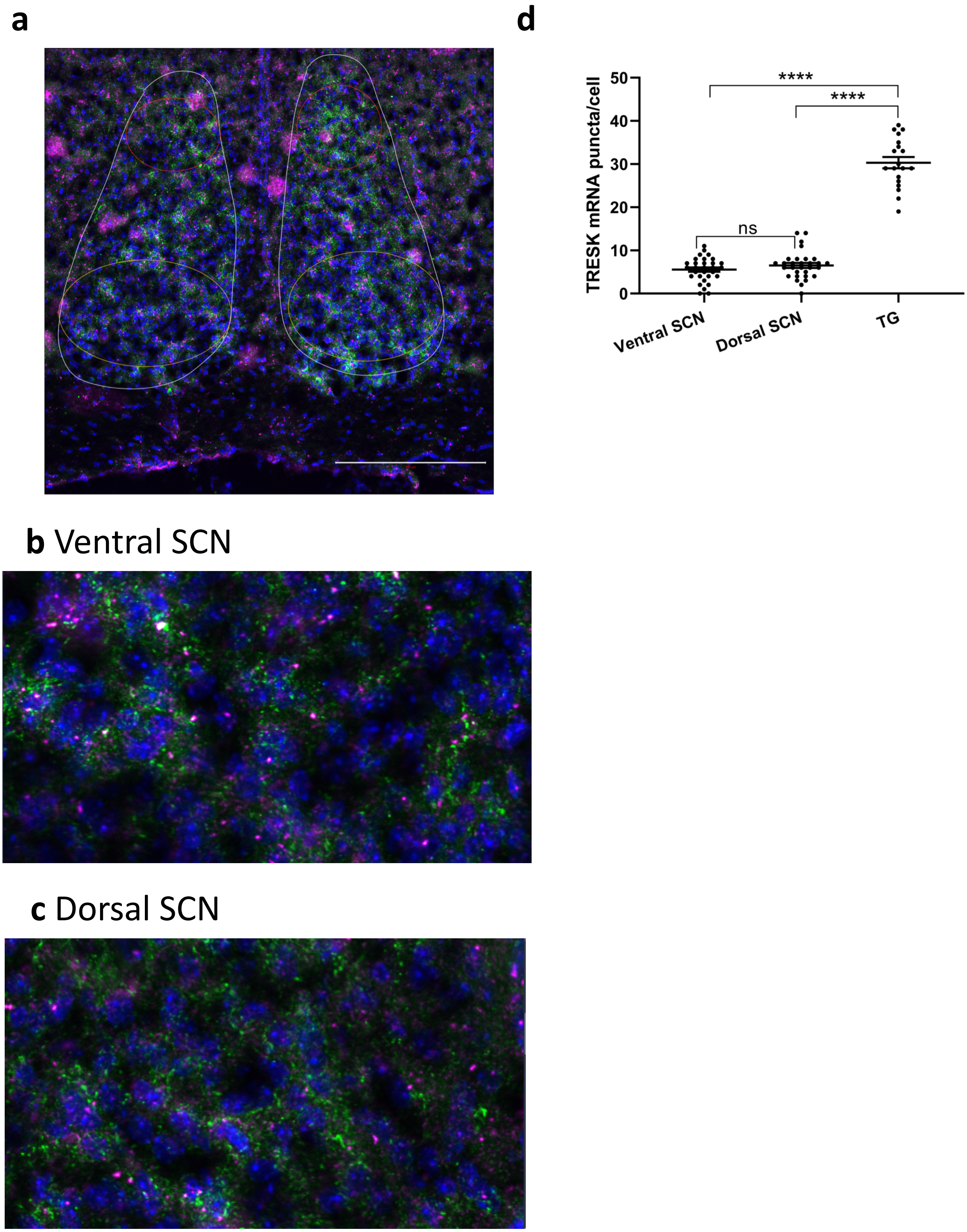

Supplementary Figure 1. *Tresk* mRNA staining in ventral vs dorsal SCN.
Figure 1a with *Tresk* mRNA staining was digitally magnified and SCN regions identified from VIP and DAPI staining, helping to identify the outline of the SCN (**a**). The lowest part of the SCN was considered ventral (**b**) and the uppermost part – dorsal **(c)**. None of the neurons from the middle of the SCN were used for analysis. Both regions of the SCN show similar distribution of *Tresk*. *Tresk* mRNA expression in TG neurons is approximately six-fold abundant compared to SCN **(d)**. One-way ANOVA, $F_{(2,\ 76)}$=291.6 p<0.0001 with Tukey's multiple comparisons test. ****p<0.0001. n=30 cells per SCN region and 20 from TG, data shown as mean ± SEM. Scale bar in a is 200µm. Staining images and puncta calculations are from one SCN section.

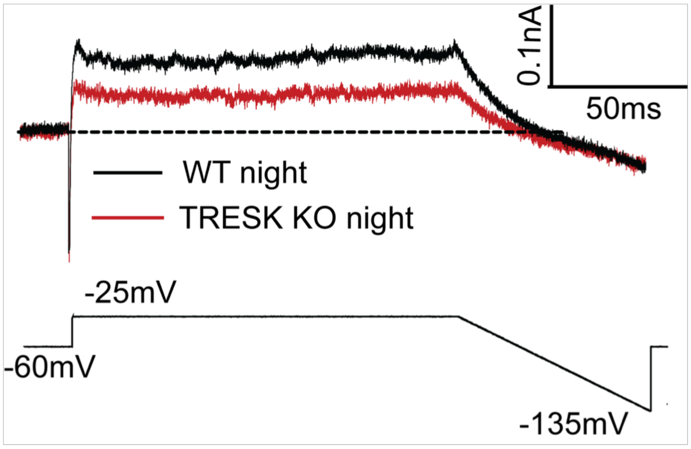

Supplementary Figure 2. Representative traces of outward potassium leak current recorded from wildtype and TRESK$^{-/-}$ SCN at night.

In voltage-clamp, SCN neurons were held at -60 mV before depolarisation to -25 mV and then subsequently ramped to -135 mV to isolate potassium leak current. Baseline measurements were collected in the presence of TTX. TRESK$^{-/-}$ had reduced nocturnal potassium leak current compared to wildtype.

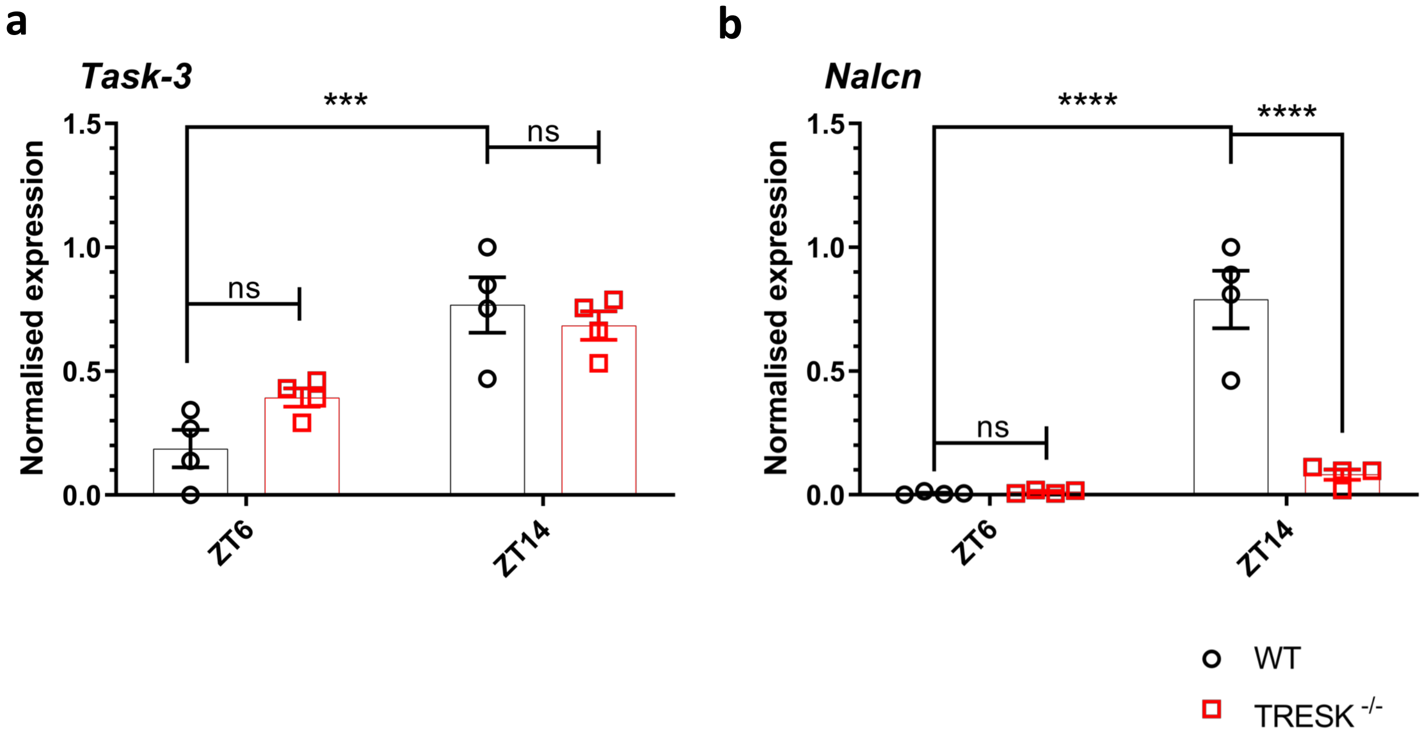

Supplementary Figure 3. Diurnal *Task-3* and *Nalcn* expression.

(a) K2P channel *Task-3* has shown diurnal expression in WT mice when measured during the day (ZT6) and night (ZT14). However, the difference of diurnal *Task-3 expression* levels were not significant in TRESK$^{-/-}$ mice, and there were no significant differences across genotypes. 2-way ANOVA with Tukey's multiple comparisons test. Significant difference were found for time of the day ($F_{(1,12)}$=33.35, p<0.0001)., but not genotype ($F_{(1,12)}$=0.6692, p=0.43). **(b)** There was a significant increase in WT *Nalcn* levels during the night, whereas TRESK$^{-/-}$did not show increased *Nalcn* expression. 2-way ANOVA was significant for all parameters: Interaction ($F_{(1,12)}$=36.58, p<0.0001), time ($F_{(1,12)}$=52.18, p<0.0001), genotype ($F_{(1,12)}$=35.31, p<0.0001) . n=4 per time point; data shown as mean ± SEM; ***p<0.001, ****p<0.0001.

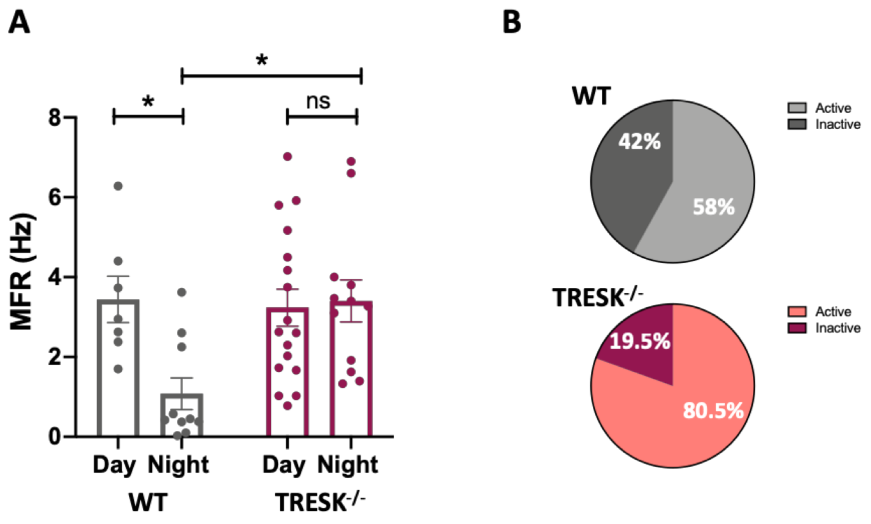

Supplementary Figure 4. Spontaneous firing rate of neurons in acute SCN slices in wildtype mice and TRESK$^{-/-}$ mice during the day and night.

A. Spontaneous firing was recorded in current clamp while zero current was inputted, showing a diurnal variation in mean firing rate (MFR) in WT but not TRESK$^{-/-}$ mice. The MFR was significantly higher in TRESK-/- mice compared to WT mice at night. Two-way ANOVA, interaction between time and genotype significant $F_{(1, 42)} = 5.628$, p=0.02) **B**. The TRESK-/- mice had an overall greater proportion of cells spontaneously firing compared to WT at night.

ns: non significant, $*p < 0.05$

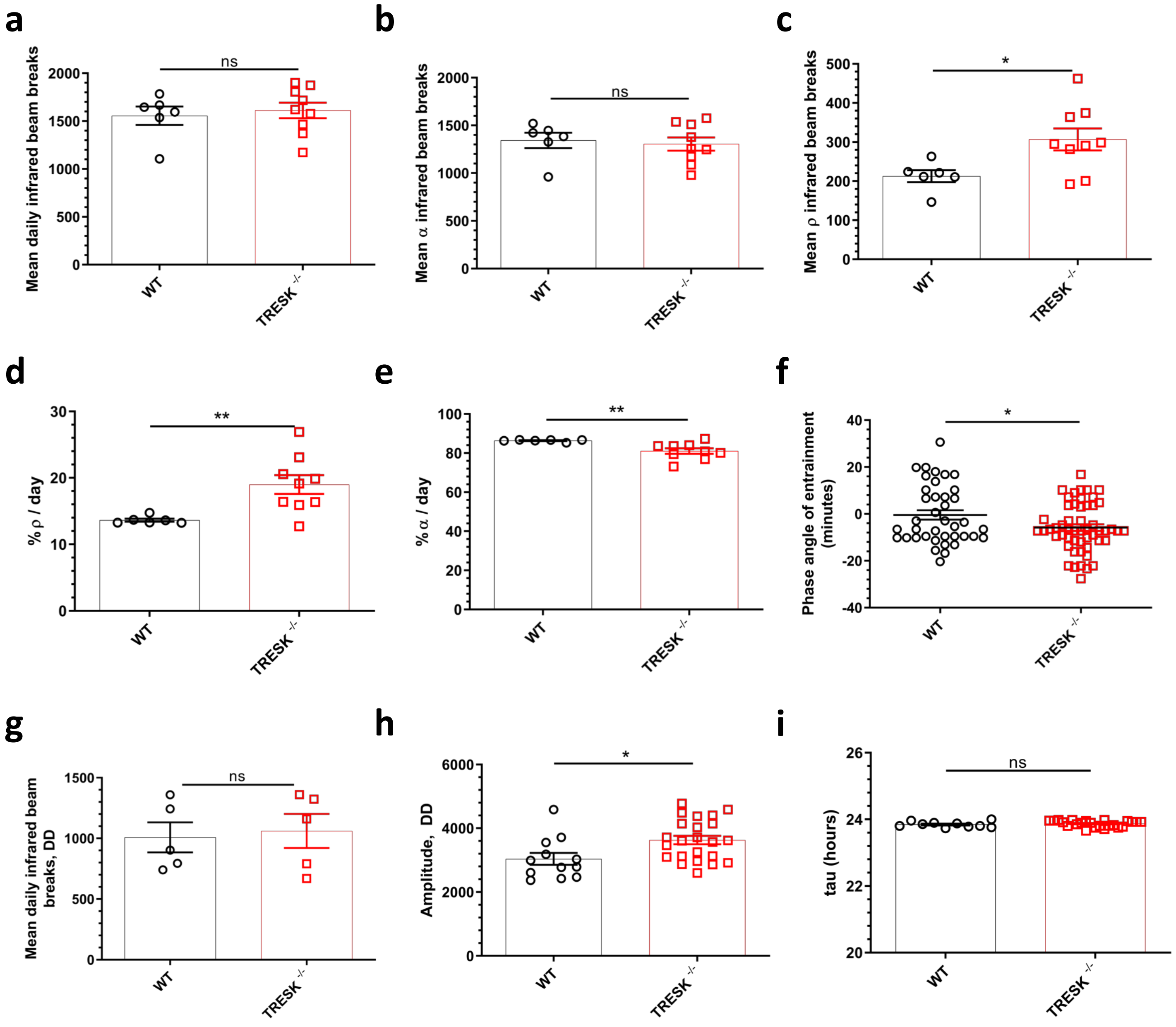

Supplementary Figure 5. Locomotor activity analysis parameters.
There are no differences in mean daily IR beam breaks between genotypes housed at 400 lux **(a)**, and in activity levels during the alpha (active) phase **(b)**, but during the inactive phase (ρ) TRESK $^{-/-}$ had significantly higher activity, p=0.025 **(c)**. The proportion daytime activity is significantly higher in the knockouts, p=0.01 **(d)**, and proportion of nighttime activity is higher in the WT, p=0.01 **(e)**. TRESK $^{-/-}$ have significantly delayed activity onset after the lights turn off, p=0.025 (phase angle of entrainment, **f**). Housing in constant dark does not affect the mean daily IR beam breaks, as there is no difference between genotypes **(g)**. TRESK $^{-/-}$ mice have more robust free-running activity, seen from the amplitude measurements, p=0.014 **(h)**, with no difference in *tau* **(i)**. All data shown as Mean ± SEM, unpaired two-tailed t-test; *<0.05, **<0.01,

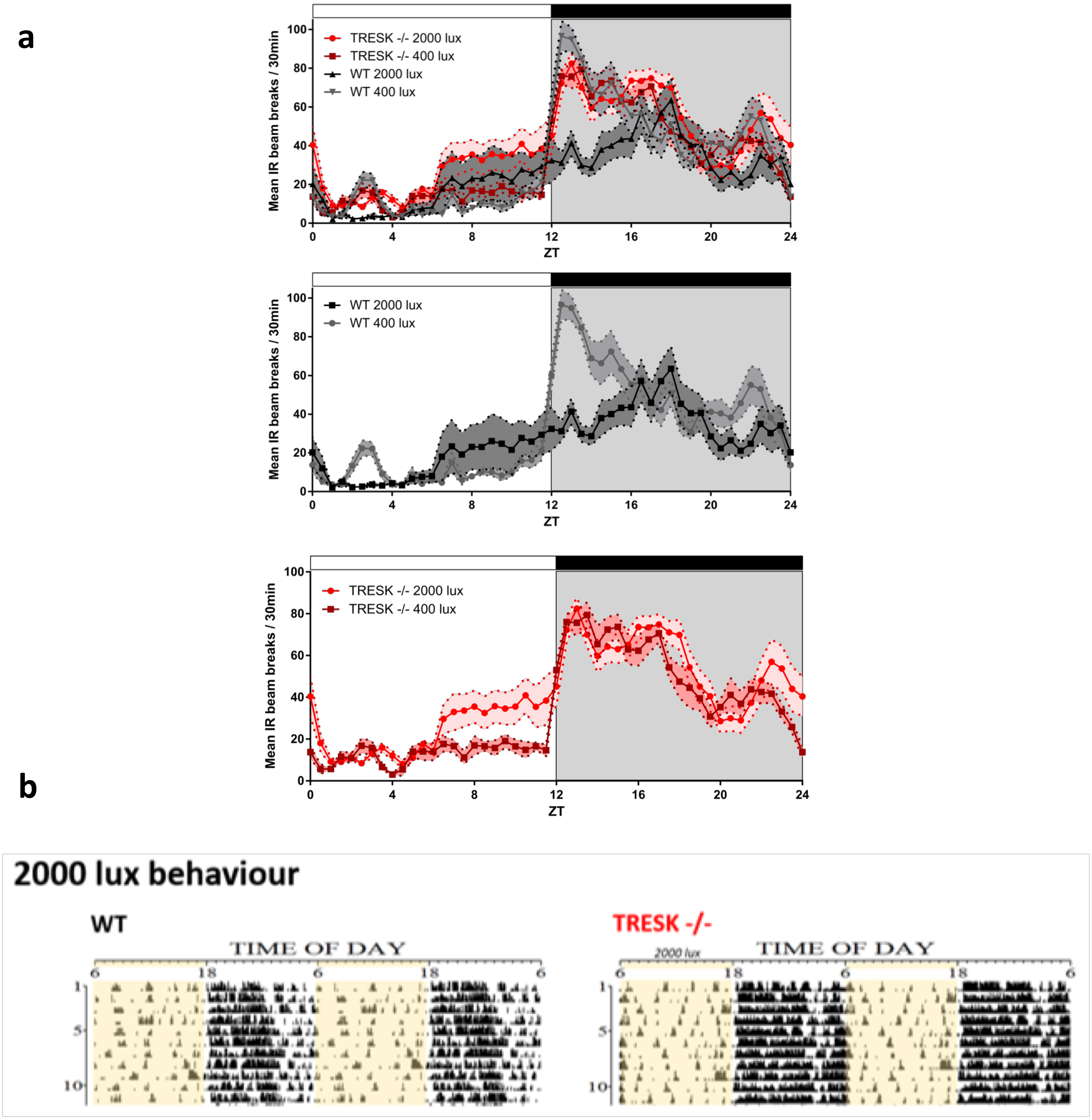

Supplementary Figure 6. Comparison of locomotor activity in WT and TRESK$^{-/-}$ mice under 400 and 2000 lux housing.
(**a**) WT animals are more active under the ambient light (middle), whereas TRESK$^{-/-}$ mice are more active under bright, 2000 lux light, compared to ambient light (bottom). From the top graph it can be seen that housing under 2000 lux caused activity suppression in WT mice. Shaded areas represent SEM. **(b)** TRESK -/- mice are more active during the second portion of the night. Behaviour was recorded using infrared sensors. 2000 lux: n= 15 (TRESK$^{-/-}$), n=7 (WT), 400 lux: n=9 (TRESK$^{-/-}$), n=6 (WT).

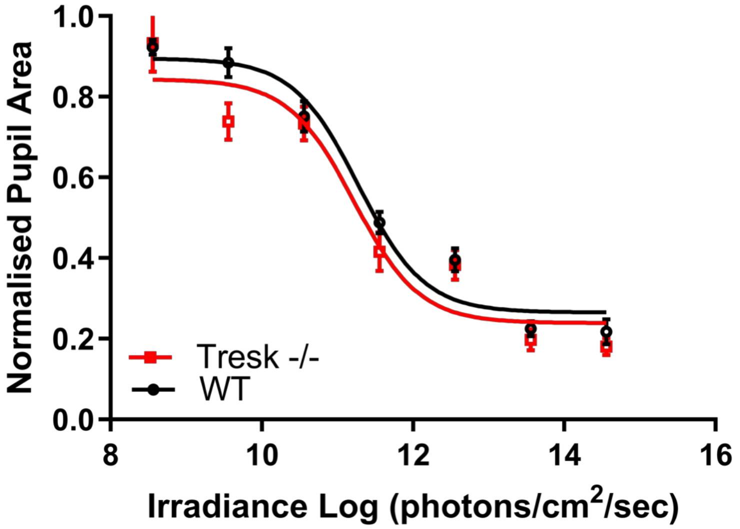

Supplementary Figure 7. Irradiance response curve (IRC) to a 480nm light stimuli.

IRC showed no significant sensitivity differences between TRESK$^{-/-}$ and WT mice between any of the irradiances indicating no deficit in retinal decoding across the visible spectrum (Bonferroni's multiple comparisons test). Two-way repeated measures ANOVA showed a significant source of variation from light intensity used ($F_{(6, 48)}$=118.4, p<0.0001) and genotype ($F_{(1, 8)}$=6.4, p<0.05), but the interaction between light intensity and genotype was not significant $F_{(6, 48)}$=0.9, p=0.5). n=5 for TRESK$^{-/-}$ and WT. All grouped data are mean ± SEM.

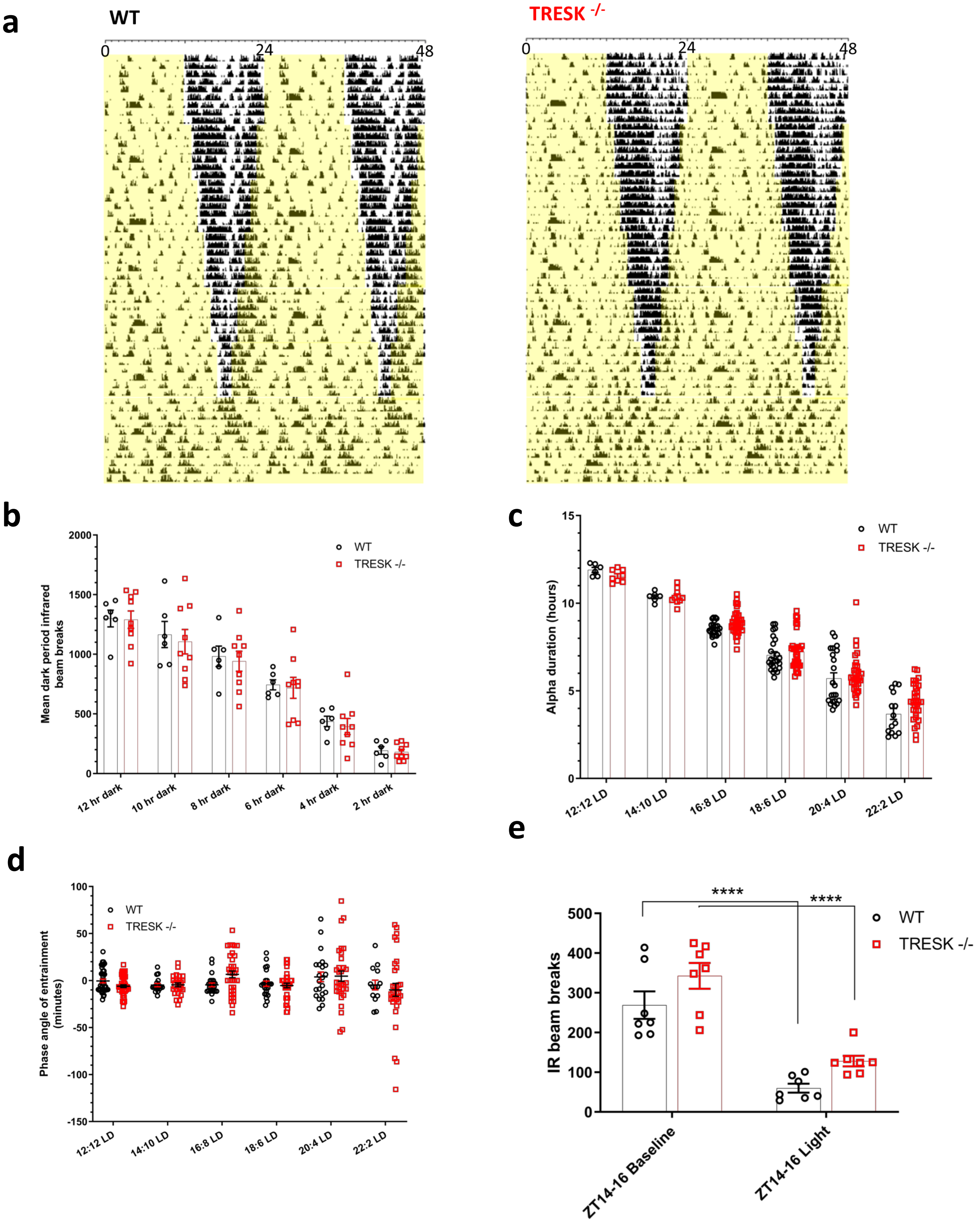

Supplementary Figure 8

Supplementary Figure 8.

(a) Representative actograms of locomotor activity during increasing photoperiods. Both, WT and $TRESK^{-/-}$ mice successfully compressed their behaviour to the dark period suggesting no changes in the mechanism of masking. Each actogram is a representative activity of one animal. Mean infrared beam breaks during the lights off period under different photoperiods showed no significant differences between genotypes (two-way ANOVA, $F_{(1, 78)}=0.5$, p=0.5) **(b)** and activity duration (alpha) in hours showed compression in activity duration in relation to deceasing dark period **(c)**. The phase angle of entrainment in minutes for each day of the measurement showed no differences between genotypes (two-way ANOVA, $F_{(1, 343)}=0.0005$, p=0.98), but which each increased photoperiod the activity onset was more and more spread within the experimental group, especially in $TRESK^{-/-}$ mice **(d)**. n=9 ($TRESK^{-/-}$), n=6 (WT).

(e) Locomotor activity during masking to 2-hour light pulses 2-4 hours following activity onset. Light pulses in the early night resulted in significant activity compression in both genotypes confirming intact masking responses. Two-way ANOVA for light ($F_{(1, 24)}=70.6$, p<0.0001), genotype ($F_{(1, 24)}=7.9$, p=0.0095); Tukey's multiple comparisons test. n=7 ($TRESK^{-/-}$), n=7 (WT). All grouped data (b-d) are mean ± SEM. **** *P < 0.0001*

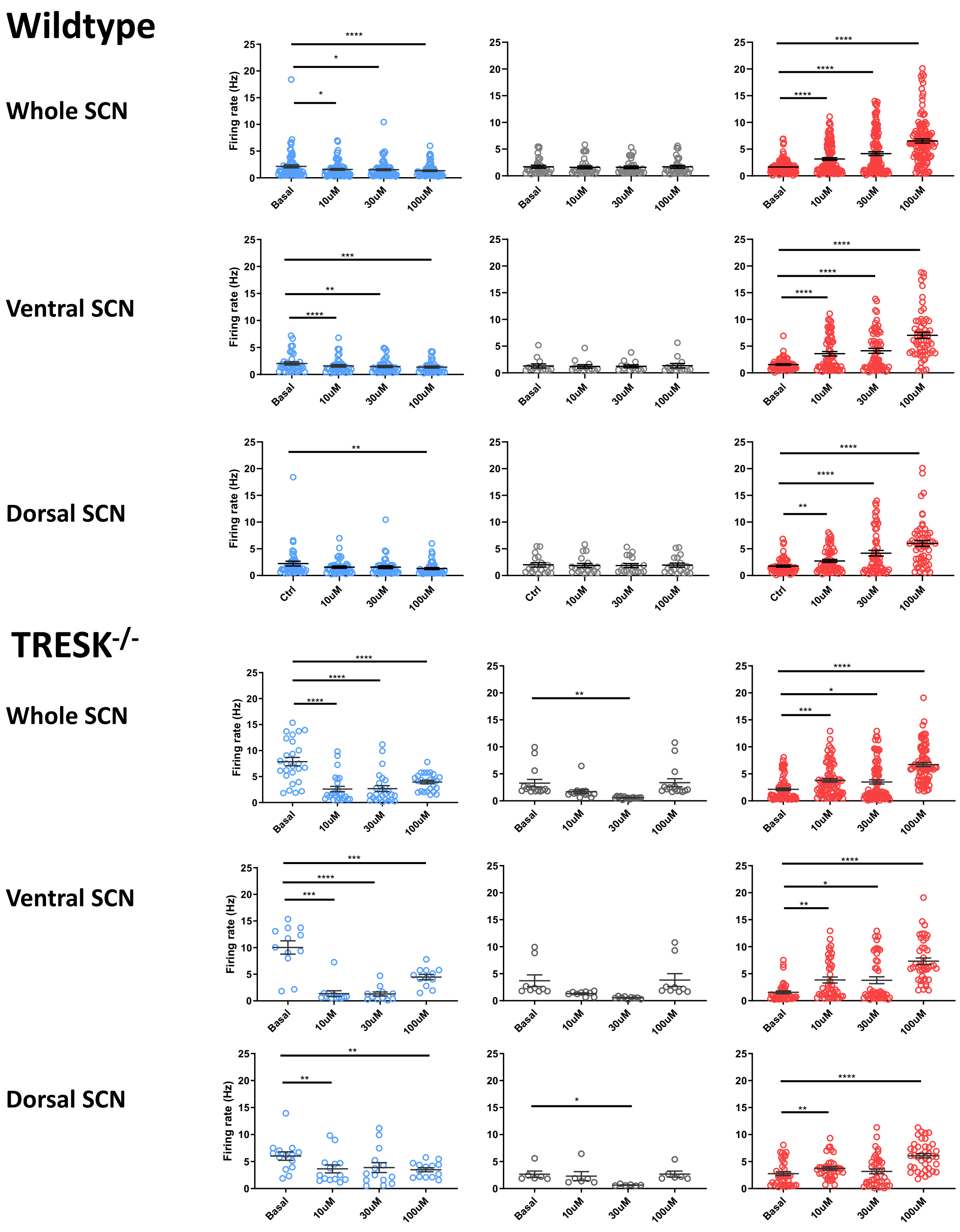

Supplementary Figure 9. The effect of glutamate on the ventral and dorsal SCN

Effect of glutamate on MFR of SCN neurons using a 256 MEA channel recording during ZT12-16 from WT or TRESK$^{-/-}$ mice (WT; day, n=7; night, n= 8 animals; TRESK$^{-/-}$; day, n = 7; night n = 5 animals). All grouped data are mean ± SEM. Activity of whole, ventral and dorsal SCN neurons in response to glutamate, by separating the MEA electrodes to those that showed reduced firing (>10% in blue), no change in activity (+/- 10% in black) and electrodes showing increased firing (>10% in red), when comparing basal and 100uM glutamate. Neurons responding to glutamate by reducing activity demonstrate a high basal MFR in TRESK$^{-/-}$ mice compared to WT. RM one-way ANOVA, p values: (Wildtype whole SCN) blue, *p=0.0404,*p=0.0215,****p=<0.0001; red, ****p=<0.0001; (Wildtype ventral SCN) blue, **p=0.0028, ***p=0.001, ****p=<0.0001; red, ****p=<0.0001; (Wildtype dorsal SCN) blue, **p=0.0082; red, **p=0.0024, ****p=<0.0001; (TRESK KO whole SCN) blue, ****p=<0.0001; black, **p=0.0056; red, *p=0.0296, ***p=0.0001, ****p=<0.0001; (TRESK KO ventral SCN) blue, ***p=0.0002, ****p=<0.0001; red, *p=0.0177, **p=0.0048, ****p=<0.0001; (TRESK KO Dorsal SCN) blue, **p=0.0062 (Basal vs 10uM), **p=0.0018 (Basal vs 100uM); black, *p=0.0342; red, **p=0.0015, ****p=<0.0001.

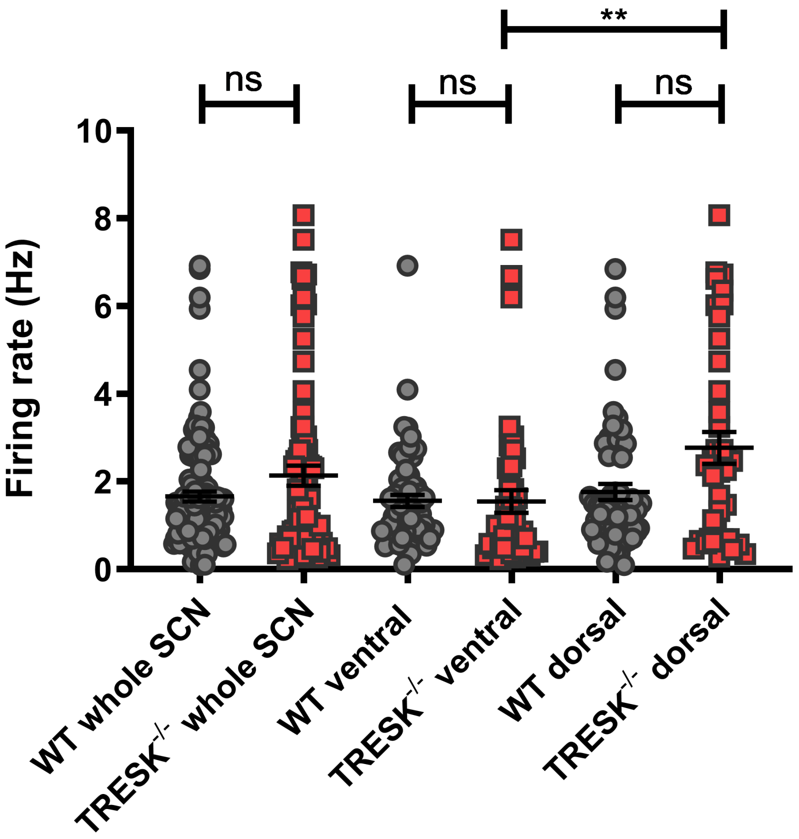

Supplementary Figure 10.

Basal SCN firing rate in electrodes that subsequently increased activity after 100uM glutamate application, in whole, ventral and dorsal SCN for WT and TRESK$^{-/-}$. ANOVA one-way (whole SCN WT and TRESK-/-) p = 0.3114, ns; ANOVA two-way log10(data) (ventral vs ventral)p = 0.4417, ns, (dorsal vs dorsal) p = 0.1855, ns, (ventral vs dorsal comparison) ***p* = 0.0044. Interaction between region and genotype significant F (1, 201) = 6.192, P=0.0136.

Supplementary Table 1.
Cosine analysis of the RT-qPCR gene expression data.

	Per1		***Per2***		***Tresk***	
	Best fit values	Standard Error	Best fit values	Standard Error	Best fit values	Standard Error
Amplitude	0.5924	0.09864	0.443	0.06394	0.4138	0.07886
Phase	-1.288	0.1955	-1.216	0.2732	-2.769	0.4372
R^2	0.6944		0.7388		0.6334	
