# Supplementary material for: TRESK is a key regulator of nocturnal suprachiasmatic nucleus dynamics and light adaptive responses
Source: Nat Commun. 2020 Sep 14;11:4614. doi: 10.1038/s41467-020-17978-9 (PMC7490422; doi:10.1038/s41467-020-17978-9)
Supplement: Supplementary file 2 — Reporting Summary [file 41467_2020_17978_MOESM2_ESM.pdf]

nature research

Corresponding author(s): Dr. Tatjana Lalic
Dr. Gurprit S. Lall
Dr. Zameel M. Cader

Last updated by author(s): 21 July 2020

Reporting Summary

Nature Research wishes to improve the reproducibility of the work that we publish. This form provides structure for consistency and transparency in reporting. For further information on Nature Research policies, see Authors & Referees and the Editorial Policy Checklist .

Statistics

For all statistical analyses, confirm that the following items are present in the figure legend, table legend, main text, or Methods section.

n/a	Confirmed	
☐	☒	The exact sample size (*n*) for each experimental group/condition, given as a discrete number and unit of measurement
☐	☒	A statement on whether measurements were taken from distinct samples or whether the same sample was measured repeatedly
☐	☒	The statistical test(s) used AND whether they are one- or two-sided<br>*Only common tests should be described solely by name; describe more complex techniques in the Methods section.*
☐	☒	A description of all covariates tested
☐	☒	A description of any assumptions or corrections, such as tests of normality and adjustment for multiple comparisons
☐	☒	A full description of the statistical parameters including central tendency (e.g. means) or other basic estimates (e.g. regression coefficient) AND variation (e.g. standard deviation) or associated estimates of uncertainty (e.g. confidence intervals)
☐	☒	For null hypothesis testing, the test statistic (e.g. *F*, *t*, *r*) with confidence intervals, effect sizes, degrees of freedom and *P* value noted<br>*Give P values as exact values whenever suitable.*
☒	☐	For Bayesian analysis, information on the choice of priors and Markov chain Monte Carlo settings
☒	☐	For hierarchical and complex designs, identification of the appropriate level for tests and full reporting of outcomes
☐	☒	Estimates of effect sizes (e.g. Cohen's *d*, Pearson's *r*), indicating how they were calculated

Our web collection on statistics for biologists contains articles on many of the points above.

Software and code

Policy information about availability of computer code

Data collection	Patch clamp data acquisition were obtained using Multiclamp 700B amplifier and the pClamp 10 software, digitized at 10-20 kHz using Digidata 1440A (Molecula Devices, CA, USA).<br>Extracellular action potential recordings (MEA) were acquired using USB-256 channel system (Multichannel Syststems, Germany).<br>Ca imaging data; the flourescence of fura2 was excited alternatively at wavelengths of 340 nm and 380 nm by means of a high speed wavelength-switching device (Multistream Pro with NI USB-60001, Cairn, UK) controlled by MetaFlour software (64 bit, v7.10.3.279, Universal Imaging, PA, USA).<br>Behaviour recordings were done using The Chronobiology Kit by Stanford Sysytems that allows the analysis of actograms.
Data analysis	Patch clamp data was analysed offline with Clampfit (pClamp10) and Neuromatic (http://neuromatic.thinkrandom.com)<br>Extracellular action potential (MEA) data was analysed using custom written scripts in Igor Pro version 6.32A and Matlab version 2017b.<br>Ca imaging analysis was analysed using image analysis software (MetaFlour, v7.10.3.279, Universal Imaging, PA, USA) and using custom scripts in Matlab version 2017b.<br>All statistical analysis and plotting was conducted using GraphPad Prism 7 software.<br>Clock gene expression over 24 hours was normalised using BioDare2 online tool by applying linear detrending. All behavioural data was extracted and processed using Microsoft Excel 2016 with data analysis and plotting performed using GrpahPad Prism 7 and 8. Image J (v1.8.0_112) was used to construct ellipses around pupil images and calculate pupil area.

For manuscripts utilizing custom algorithms or software that are central to the research but not yet described in published literature, software must be made available to editors/reviewers. We strongly encourage code deposition in a community repository (e.g. GitHub). See the Nature Research guidelines for submitting code & software for further information.

Data

Policy information about availability of data

All manuscripts must include a data availability statement. This statement should provide the following information, where applicable:

- Accession codes, unique identifiers, or web links for publicly available datasets
- A list of figures that have associated raw data
- A description of any restrictions on data availability

The data sets generated during and/or analysed during the current study are available from the corresponding author upon reasonable request.

Field-specific reporting

Please select the one below that is the best fit for your research. If you are not sure, read the appropriate sections before making your selection.

[x] Life sciences [ ] Behavioural & social sciences [ ] Ecological, evolutionary & environmental sciences

For a reference copy of the document with all sections, see nature.com/documents/nr-reporting-summary-flat.pdf

Life sciences study design

All studies must disclose on these points even when the disclosure is negative.

Sample size	The sample size was chosen based based on the pilot studies and consistent to data presented in previous publications. For behavioural experiments, sample sizes were calculated using G*Power Cohen's d effect size comparison at > 80% power with significance at alpha =0.05. For electrophysiological experiments, no statistical methods were used to predetermine sample sizes. However, sample sizes used in the present study were similar to previous studies using in vitro electrophysiological recordings (Threlfell et al., Weir et al., Pettingill et al.,). Sample sizes adopted in this study were sufficient for detecting robust effect.
Data exclusions	In the MEA analysis, inactive electrodes were excluded in the analysis. Data was only excluded if there was an error when undertaking a given protocol, otherwise no data was excluded.
Replication	In all experiments both positive and negative controls formed part of the design, thus allowing replication and verification of our data. All experiments were conducted on individual animals all derived from heterozygous breeding pairs to generate age matched experimental and control animals. Throughout the entire study, at least three replicates of each experiment were undertaken and appropriately analysed for statistical significance.
Randomization	A randomized within subjects design was employed in our behavioral studies. For all behavioral experiments mice were randomly allocated and randomly placed under the infrared sensors with a mix of genotypes in each row to reduce any light or housing position effects on locomotor activity.
Blinding	All behavioral analysis was undertaken blind by two investigators, with treatment groups disclosed post data collection. There were no discrepancies between data sets analysed by the two investigators. It was not always possible to blind electrophysiological experiemnts due to following fixed procedures, however, all data analysis was performed blind.

Reporting for specific materials, systems and methods

We require information from authors about some types of materials, experimental systems and methods used in many studies. Here, indicate whether each material, system or method listed is relevant to your study. If you are not sure if a list item applies to your research, read the appropriate section before selecting a response.

Materials & experimental systems

n/a	Involved in the study	
[ ]	[x]	Antibodies
[x]	[ ]	Eukaryotic cell lines
[x]	[ ]	Palaeontology
[ ]	[x]	Animals and other organisms
[x]	[ ]	Human research participants
[x]	[ ]	Clinical data

Methods

n/a	Involved in the study	
[x]	[ ]	ChIP-seq
[x]	[ ]	Flow cytometry
[x]	[ ]	MRI-based neuroimaging

Antibodies

Antibodies used	Rabbit polyclonal anti-VIP antibody (Abcam, ab43841); Goat Anti-Rabbit Alexa Fluor 488, (Invitrogen A11008, lot 1937184.
Validation	Tested application by abcam IHC-FoFr, ICC/IF, IHC-Fr; Species reactivity with: Mouse, Rat, Pig, Zebrafish. Please note this antibody has been discontinued and replaced by ab8556.

Animals and other organisms

Policy information about studies involving animals; ARRIVE guidelines recommended for reporting animal research

Laboratory animals	This study involved wild type mice, C57BL/6J strain and TRESK knockout mice. TRESK (GenBank accession number NM332396, Ensembl identification number ENSMUSG40901) knockout mice were obtained from the KOMP repository. TRESK knockout mice were initially crossed with wild-type C57Bl/6J (Harlan, United Kingdom). Offspring from F5 heterozygous littermates were used in this study. All mice were group housed and only individually caged for behaviour assessment. All holding and experimental animal rooms were in the temperature range 19-24 degrees celsius and humidity 50% (+/- 15) regulated in accordance with UK Home office guidelines.<br>All electrophysiology data was collected from P16-P23 wildtype C57BL/6 strain and TRESK-/- mice of either sexes. Mice were housed at the University of Oxford in holding rooms on a 12/12-h light/dark cycle (lights on at 07:00 h and off at 19:00 h) in single-sex groups of 2–6 with ad libitum food and water.<br><br>All reported behavioral and gene expression data was performed on wildtype C57BL/6 and TRESK-/- male mice up to 1 year old. All behavioural testing was performed at Charles River Animal Facility using infrared sensors (LuNAR™ PIR 360°, Risco Goup) and the data was collected using The Chronobiology Kit (Stanford Software systems). Male mice were housed individually in polypropylene cages with food and water available ad libitum.<br>All procedures complied with the UK Animals (Scientific Procedures) Act (1986) and were performed under a UK Home Office Project Licence in accordance with University of Oxford and University of Kent Policy on the Use of Animals in Scientific Research. This study conforms to the ARRIVE guidelines.
Wild animals	
Field-collected samples	The study did not involve wild animals.
Ethics oversight	The study did not involve samples collected from the field.<br><br>This study was carried out in accordance with the principles of the United Kingdom Home Office Animals in Scientific Procedures Act (1986). The protocol was approved by the clinical medicine animal care and ethical review body, University of Oxford and University of Kent Policy on the Use of Animals in Scientific Research.

Note that full information on the approval of the study protocol must also be provided in the manuscript.
